# Antiaging Potential of Lipophilic Extracts of *Caulerpa prolifera*

**DOI:** 10.3390/md23020083

**Published:** 2025-02-14

**Authors:** Gonçalo P. Rosa, Maria Carmo Barreto, Ana M. L. Seca, Diana C. G. A. Pinto

**Affiliations:** 1 University of the Azores, Faculty of Sciences and Technology, Centre for Ecology, Evolution and Environmental Changes (cE3c), Azorean Biodiversity Group & Global Change and Sustainability Institute (CHANGE), 9501-321 Ponta Delgada, Portugal; goncalo.p.rosa@uac.pt (G.P.R.); maria.cr.barreto@uac.pt (M.C.B.); ana.ml.seca@uac.pt (A.M.L.S.); 2 LAQV-REQUIMTE, Department of Chemistry, University of Aveiro, Campus Universitário de Santiago, 3810-193 Aveiro, Portugal

**Keywords:** macroalgae, phytochemical profile, cosmeceutical potential

## Abstract

The cosmeceutical industry has increasingly turned its attention to marine macroalgae, recognizing their significant bioactive potential as sources of natural compounds for skincare applications. A growing number of products now incorporate extracts or isolated compounds from various macroalgae species. However, many species remain underexplored, highlighting a valuable opportunity for further research. Among these, *Caulerpa prolifera* (Forsskål) J.V. Lamouroux has emerged as a promising candidate for cosmeceutical applications. This study provides the most comprehensive phytochemical assessment of *C. prolifera* to date, revealing its potential as a source of bioactive extracts and compounds. The analysis identified key components of its lipophilic profile, predominantly saturated and unsaturated fatty acids, alongside di-(2-ethylhexyl) phthalate—an endocrine disruptor potentially biosynthesized or bioaccumulated by the algae. While the crude extract exhibited moderate tyrosinase inhibitory activity, its overall antioxidant capacity was limited. Fractionation of the extract, however, yielded subfractions with distinct bioactivities linked to changes in chemical composition. Notably, enhanced inhibitory activities against elastase and collagenase were observed in subfractions enriched with 1-octadecanol and only traces of phthalate. Conversely, antioxidant activity diminished with the loss of specific compounds such as β-sitosterol, erucic acid, nervonic acid, and lignoceric acid. This work advances the understanding of the relationship between the chemical composition of *C. prolifera* and its bioactivities, emphasizing its potential as a source of cosmeceutical ingredients, leading to a more comprehensive valorization of this macroalga.

## 1. Introduction

Skin aging is a multifaceted and dynamic process influenced by a combination of intrinsic and extrinsic factors. Intrinsic factors include genetic predisposition, natural metabolic changes, and hormonal fluctuations that occur with age, while extrinsic factors primarily involve environmental influences such as UV radiation; pollution; and lifestyle habits like smoking, diet, and stress. Together, these factors lead to cumulative damage at the cellular and molecular levels, resulting in visible signs of aging, including wrinkles, loss of skin elasticity, dehydration, and pigmentation irregularities [1,2,3,4,5]. The complexity of these processes has resulted in a continuous search for effective strategies to mitigate the effects of aging, with natural compounds emerging as a promising avenue for intervention [6].

The search for effective anti-aging compounds has garnered significant attention in recent years due to the growing demand for products that mitigate the visible and functional signs of skin aging. Oxidative stress, caused by an imbalance between the production of reactive oxygen species (ROS) and the skin’s antioxidant defenses, is one of the primary drivers of aging. ROS can lead to cellular damage, lipid peroxidation, and degradation of the extracellular matrix (ECM), accelerating the aging process. Therefore, studying the antioxidant potential of compounds is crucial, as these molecules can neutralize ROS and protect the skin from oxidative damage [5,7].

In addition to their antioxidant properties, compounds with metal-chelating activity are also of great interest. Metal ions, such as iron and copper, can catalyze the formation of ROS through Fenton-type reactions, exacerbating oxidative stress and tissue damage. Chelating agents bind to these metal ions, reducing their availability to participate in harmful reactions and providing an additional mechanism to combat skin aging [8,9].

Furthermore, the inhibition of enzymes such as elastase, collagenase, hyaluronidase, and tyrosinase is a critical parameter in evaluating anti-aging potential. Elastase and collagenase degrade key ECM proteins, such as elastin and collagen, leading to the formation of wrinkles and loss of skin elasticity [3]. Hyaluronidase breaks down hyaluronic acid, an essential molecule for maintaining skin hydration and volume, contributing to dryness and reduced firmness [10]. Tyrosinase, involved in melanin synthesis, plays a central role in hyperpigmentation, which is often associated with aged skin [11]. By targeting these enzymes, bioactive compounds can preserve the structural and functional integrity of the skin, reduce signs of aging, and improve overall skin health.

In this context, the cosmeceutical industry has shown growing interest in macroalgae-based products due to their remarkable antiaging properties. These properties include potent antioxidant activities that combat oxidative stress, anti-inflammatory effects that reduce skin irritation, and the ability to protect and maintain the extracellular matrix, which is essential for skin structure and resilience [6,12]. Additionally, macroalgae-derived compounds are valued for their low cytotoxicity and reduced allergen content when compared to synthetic alternatives, making them safer and more appealing for consumers seeking effective yet gentle skincare solutions [13,14,15,16,17]. As a result, macroalgae have gained recognition as a powerful and versatile resource for the development of innovative cosmeceuticals that promote skin health and address the multifactorial challenges of aging.

Among the diverse array of macroalgae with bioactive potential, *Caulerpa prolifera* (Forsskål) J.V. Lamouroux stands out as a promising candidate. It is widely distributed in tropical and subtropical waters, including the Caribbean Sea and the Gulf of Mexico. It is also the only native *Caulerpa* species in Europe and can be found in the Mediterranean Sea as well as in subtropical and tropical regions of the Atlantic Ocean [18]. Although it is considered native to the regions mentioned above, *C. prolifera* has become invasive in various other habitats. The westernmost recorded occurrence of this species in European waters is in the Azores Islands, located in the northeastern Atlantic [19].

Extracts of different polarities from *C. prolifera* have demonstrated significant antimicrobial activity, inhibiting the growth of both gram-positive and gram-negative marine bacteria [20]. Additionally, studies by Abdel-Wahhab et al. [21] have investigated the hepatoprotective properties of an aqueous extract of *C. prolifera* against aflatoxin B1-induced hepatotoxicity in female Sprague Dawley rats. Remarkably, pre-treatment with the aqueous extract of *C. prolifera* resulted in improvements across all tested parameters, indicating its hepatoprotective effectiveness.

Additionally, Costa et al. [22] revealed that sulfated polysaccharides extracted from *C. prolifera* exhibit anti-proliferative effects, inhibiting cell proliferation by approximately 57% at a concentration of 0.1 mg/mL. Moreover, another subfraction of sulphated polysaccharides demonstrated high osteogenic potential, enhancing alkaline phosphatase activity, and promoting the accumulation of calcium in the extracellular matrix without exhibiting toxicity against hMSCs-WJ cells at concentrations up to 10 mg/mL after 72 h of exposure. Furthermore, it was recently found that an extract rich in sulphated polysaccharides from *C. prolifera* is capable of suppressing lipid accumulation both in vitro and in vivo [23], showing the interest in this class of compounds.

Methanolic extracts of *C. prolifera* have been found to possess significant lipoxygenase inhibition properties [24]. Furthermore, these extracts were observed to elevate the levels of antioxidant enzymes in the liver of *Coris julis*, with activities attributed to the presence of caulerpenyne within the extract [25].

Caulerpin, a characteristic marine alkaloid with potent antioxidant and anti-inflammatory activities, which has been studied for its role in mitigating oxidative stress and modulating inflammatory pathways, has also been identified in *C. prolifera* [26,27,28]. Furthermore, *C. prolifera* produces sesquiterpenoids, such as furocaulerpin, which has demonstrated bioactivities including cytotoxicity against cancer cell lines and antioxidant effects, highlighting their therapeutic potential [29,30]. Squalene, a highly unsaturated triterpenoid, is recognized for its exceptional antioxidant activity and protective effects against oxidative stress-induced damage as well as its ability to enhance skin lipid composition and prevent moisture loss [31].

The literature indicates that the chemical composition of *C. prolifera* is influenced by seasonality, driven by variations in environmental factors such as temperature, nutrient availability, salinity, and light exposure, among others. This variability should be carefully considered when proposing the species for industrial applications, as it may impact the consistency and efficacy of its bioactive compounds [32,33].

This multifaceted range of biological activities highlights the diverse pharmacological potential of *C. prolifera*. Despite its potential, this species remains largely underexplored regarding its cosmeceutical potential, which shows an important gap. In this regard, the aim of the present work is to bridge this gap by studying the phytochemical composition of *C. prolifera* with a focus on its lipophilic profile and key bioactive compounds and to evaluate its anti-aging potential through the inhibition of enzymes such as tyrosinase, elastase, and collagenase. Additionally, this study aimed to fractionate the crude extract to link variations in chemical composition to its antioxidant and enzymatic inhibition capacity and explore its overall potential as a source of cosmeceutical ingredients.

## 2. Results and Discussion

### 2.1. Extracts Preparation

The extraction of *C. prolifera* biomass was performed by maceration, sequentially increasing the polarity of the extraction solvents. The extraction process resulted in the preparation of three extracts with distinct polarity, and the masses obtained for the respective extraction yields are presented in Table 1.

This table illustrates the extract yields resulting from sequential extraction of *C. prolifera* dry biomass using different solvents, with dichloromethane yielding the highest amount of extract. In comparison, extraction with acetone resulted in a significantly lower yield, nearly eight times less than dichloromethane and three times less than ethanol. The better results obtained with dichloromethane suggest that the *C. prolifera* biomass presents a higher amount of less polar compounds which are more readily extracted by this solvent. Other works containing information about the extraction yield of this species are scarce and not comparable to this study due to differing extraction parameters. A study on *C. racemosa* and *C. lentilifera* reported higher yields for chloroform (5.2% and 5.1%, respectively) and methanol (12.6% and 13.5%, respectively) extracts, obtaining higher yields with the more polar solvent [34], which is contrary to the observations in the present work. However, it should be noted that in that study, the extraction was not sequential.

### 2.2. Bioguided Fractionation of the Extracts

#### 2.2.1. Biological Activities of the Extracts

**Figure 1:**
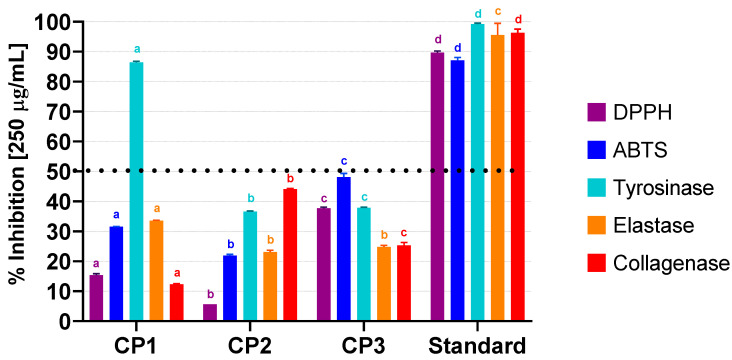
Assessment of the antioxidant and enzymatic inhibition activities of *C. prolifera* extracts (250 µg/mL) as determined by the DPPH and ABTS scavenging assays and by the inhibition of tyrosinase, elastase, and collagenase assays, respectively. Results are presented as % of radical scavenged for DPPH and ABTS and as % of enzyme inhibition for the enzymatic assays. The standard compounds were Trolox for DPPH and ABTS, kojic acid for tyrosinase, EDTA for collagenase (all at 250 µg/mL), and N-methoxysuccinyl-Ala-Ala-Pro-chloromethylketone (at 10 µg/mL) for elastase. Different letters above the bars indicate statistically significant differences (*p* < 0.05) within each assay. Bars sharing the same letter within the same assay are not significantly different.

A bioguided fractionation approach was employed to identify compounds produced by *C. prolifera* with potential cosmeceutical applications. The three extracts obtained were tested for their antioxidant and chelating activities. Additionally, their effects on enzymes associated with antiaging properties, namely tyrosinase, elastase, collagenase, and hyaluronidase, were assessed, with the results being presented in Figure 1 (numerical data can be found in Tables S1 and S2).

Oxidative stress is a key contributor to skin aging, primarily through the production of reactive oxygen species (ROS). ROS cause cellular damage, lipid peroxidation, and degradation of the extracellular matrix (ECM), leading to visible signs of aging.

Additionally, the inhibition of enzymes such as elastase, hyaluronidase, and tyrosinase provides insights into a compound’s anti-aging potential. Elastase contributes to skin flaccidity by degrading elastin, while hyaluronidase accelerates dehydration and reduces skin volume through the breakdown of hyaluronic acid. Tyrosinase drives hyperpigmentation, leading to an uneven skin tone. Together, oxidative stress and enzymatic activity exacerbate the formation of wrinkles, loss of elasticity, and other hallmarks of aging, making the evaluation of these mechanisms essential for identifying effective anti-aging agents.

None of the extracts presented an antioxidant effect by the chelating process or hyaluronidase inhibitory activity at the maximum concentration tested (250 µg/mL), so these assays were not included in Figure 1.

The results for the antioxidant activity show that CP1 exhibited a low DPPH radical scavenging activity at the maximum concentration tested, with an antioxidant activity (AA%) of 15.3% ± 0.54, while CP2 showed a lower AA% of 5.6% ± 0.01. CP3 displayed the highest DPPH scavenging activity with an AA% of 37.7% ± 0.37. With a variation in the same direction, in the ABTS assay, CP1, CP2, and CP3 exhibited AA% values of 31.5% ± 0.11, 21.9% ± 0.37, and 48.1% ± 1.21, respectively. However, all these results were significantly lower than the activity exhibited by Trolox, a well-known antioxidant compound, that showed robust radical scavenging activity in the DPPH assay, with an AA% of 89.7% ± 0.50 at the maximum concentration tested of 250 µg/mL and an IC_50_ of 7.3 ± 0.09 µg/mL. Similarly, in the ABTS assay, Trolox displayed an AA% of 87.1% ± 0.95 and an IC_50_ of 2.68 ± 0.08 µg/mL. The low antioxidant activity of *C. prolifera* was already suggested by Chaabani et al. [35] who described the extracts obtained by two different extraction methods as inactive.

Results published concerning the antioxidant activity of other *Caulerpa* species are very variable. One work reported that *C. lentilifera* extracts had EC_50_ of 2.20, 9.74, and 81.55 mg/mL for chloroform, methanol, and water extracts, respectively [34], and these values were much higher than the maximum concentration tested in the present work. Actually, EC_50_ values on the order of mg/mL cannot be interpreted as excellent results, contrary to the statement by the authors.

The same authors [34] reported for *C. racemosa* an EC_50_ of 0.65 mg/mL for the chloroform extract, 2.51 for the methanolic extract, and 7.46 for the aqueous extract. Interestingly, for both these species, the non-polar extracts were more active than the polar ones, contrary to what was observed in the present work.

Another study reported that, for the same macroalgae (*C. racemosa*), an ethanolic extract presented EC_50_ of 52.84 and 79.73 µg/mL for DPPH and ABTS assays, respectively [36], and another study with the same species indicated an ethanolic extract obtained by maceration with an EC_50_ of 49.0 ± 4.0 μg/mL on the DPPH assay [37]. This difference in results for the same species shows the importance of the location where the macroalgae is collected on the bioactivities it presents. Furthermore, the results can be influenced by the seasonality of the species and by using different solvents and extraction methodologies that can significantly affect the extraction, as demonstrated with other macroalgae species [38].

Regarding the enzymatic assays, CP1 exhibited inhibitory effects on tyrosinase, inhibiting 86.4% ± 0.38 of enzyme activity at 250 µg/mL, which allowed for the determination of an IC_50_ = 31.3 ± 0.37 µg/mL. This value was only 17.2 times higher than the one obtained for the standard compound, kojic acid (IC_50_ = 1.82 ± 0.13 µg/mL), which is remarkable for an extract containing a wide array of compounds.

It is even more remarkable when compared with results obtained for other *Caulerpa* species. The ethanolic extracts of *C. lentilifera* and *C. racemosa* were inactive against this enzyme, even at a concentration of 5 mg/mL [39], which is 20 times more concentrated than the maximum concentration tested in this work. Furthermore, CP1 is also more active against tyrosinase than the ethanolic extracts of other green macroalgae, like *Ulva rigida* (IC_50_ > 5000 µg/mL) and *Ulva intestinalis* (IC_50_ = 3350 ± 0.12 µg/mL) [39].

For elastase inhibition, CP1 demonstrated a moderate inhibitory effect, with a percentage inhibition of 33.5% ± 0.21 at 250 µg/mL. CP2 and CP3 exhibited lower inhibitory effects, with percentages of 23.1% ± 0.58 and 24.8% ± 0.52, respectively. In fact, there are no reports about elastase inhibition by *Caulerpa* species. Moreover, results for green macroalgae concerning this enzyme are scarce and not very promising, as in the case of *Ulva lactuca*, where hot water extracts presented no elastase inhibition [40]. This may indicate a low potential to find good elastase inhibitors on this group of macroalgae or that most works did not study elastase inhibition.

Regarding collagenase inhibition, the extract CP2 was the most active extract at 250 µg/mL, inhibiting 44.1 ± 0.21% of the enzymatic activity, followed by CP3 (25.3% ± 0.98) and CP1 (12.3% ± 0.22). Since collagenase is a metalloproteinase, it requires a metallic cofactor to exert its functions. Interestingly, none of the extracts exhibited chelating activity, suggesting that the observed levels of collagenase inhibition are not attributable to the quenching of the metallic cofactor. This is, as far as could be determined, the first report of the collagenase inhibition potential in the *Caulerpa* genus.

Since CP1 was the extract that showed more promise, especially regarding tyrosinase inhibition, it was selected to be chemically characterized, aiming to identify the compounds responsible for those bioactivities.

#### 2.2.2. GC-MS Analysis

**Figure 2:**
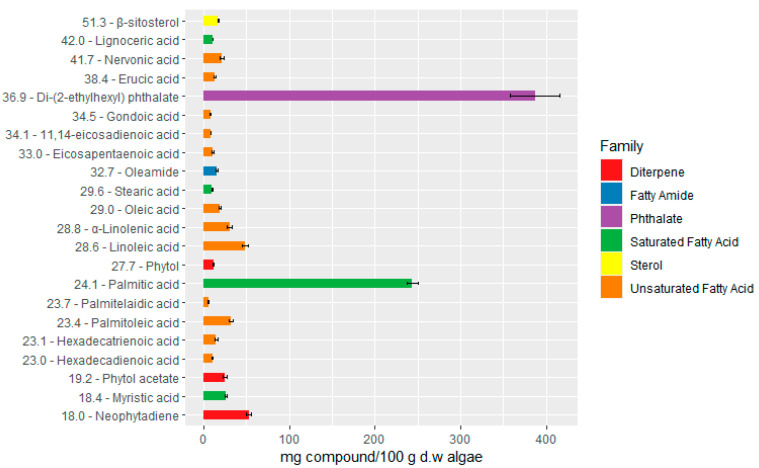
Compounds identified by GC/MS in the dichloromethane extract of *C. prolifera* (CP1). The numbers before the name of the compound are the retention time (Rt) in minutes.

The chemical composition of the dichloromethane extract from *C. prolifera* (CP1) was analyzed using GC-MS. This analysis facilitated the identification of several volatile metabolites belonging to different families, with hydroxylated and carboxylated compounds being identified as TMS derivatives. The identification of most peaks was conducted by matching peaks with entries in the GC-MS spectral library and with MS data found in the literature. Additionally, the retention times and mass spectra were compared with those of pure standards injected under the same experimental conditions. The quantitative analysis was also performed, and the numerical values obtained and used to construct the graph in Figure 2 are those shown in Table S3 in the Supplementary Material.

**Figure 3:**
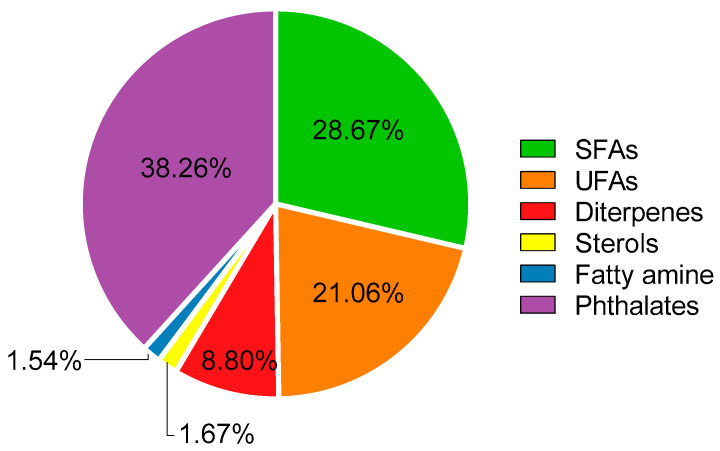
Relative abundance of each family of compounds present in CP1 extract.

The GC-MS analysis of CP1 showed the presence of 22 different compounds dominated by the presence of palmitic acid and a phthalate (Figure 2), comprising approximately 23.9% and 37.9% of the total mass of identified compounds, respectively. The results presented in Figure 2 indicate a prominent presence of saturated (SFA), monounsaturated (MUFA), and polyunsaturated (PUFA) fatty acids (Figure 3) reveals that a significant portion of the identified compounds—specifically, 49.73%—corresponded to fatty acids, with saturated fatty acids representing the biggest portion of total fatty acids (28.67%). Palmitic acid was the most abundant (243.70 mg/100 g d.w.), followed by myristic acid (26.15 mg/100 g d.w.), lignoceric acid (11.07 mg/100 g d.w.), and stearic acid (9.74 mg/100 g d.w.). These fatty acids possess inherent emollient properties, aiding skin hydration and providing a protective barrier against environmental stressors. Moreover, recent studies have highlighted their involvement in regulating skin lipid composition and influencing the expression of key proteins involved in epidermal barrier function [41,42]. Some saturated fatty acids, such as palmitic, stearic, and lauric acids, have been reported to exhibit moderate anti-tyrosinase activity, although their activity is generally less potent compared to unsaturated fatty acids [43,44]. Nevertheless, their presence in the extract enhances its potential as an anti-hyperpigmentation agent by contributing to the overall tyrosinase inhibition observed, particularly when combined with other bioactive compounds that may act synergistically. This highlights the extract’s suitability for applications targeting skin pigmentation disorders. The reported presence of these compounds in other studies reinforces their potential role in the observed bioactivities of *C. prolifera*, supporting its cosmeceutical applications [45,46]. The abundance of SFA in the extract indicates a great potential for *C. prolifera* as a source of components for cosmetic formulations.

Furthermore, alongside the presence of saturated fatty acids (SFAs), the CP1 extract also revealed the presence of unsaturated fatty acids, accounting for 21.06% of the total identified compounds. While the proportion of unsaturated fatty acids (UFAs) was lower compared to saturated fatty acids (SFAs), the diversity within UFAs was broader, with the identification of 11 distinct compounds, contrasting the four SFAs detected (Figure 2). Within the UFA group of compounds, 44.70% corresponded to monounsaturated fatty acids (MUFAs), with the remaining 55.3% being polyunsaturated fatty acids (PUFAS). The most abundant ones were linoleic acid (48.22 mg/100 g d.w.), palmitoleic acid (32.28 mg/100 g d.w.), and a-linolenic acid (30.22 mg/100 g d.w.).

Unsaturated fatty acids, particularly PUFAs, exert important regulatory roles in modulating inflammatory cascades and preserving cutaneous homeostasis. α-Linolenic acid, for instance, serves as a precursor for the synthesis of anti-inflammatory lipid mediators, such as resolvins and protectins, which actively mitigate inflammation and facilitate tissue regeneration [47,48,49]. Additionally, its participation in the structural integrity of the stratum corneum and its influence on sebaceous gland activity render it indispensable in formulations tailored for acne-prone and sebum-regulating skincare regimens [50]. The seasonal variability in PUFA content reported for *C. prolifera* in previous studies highlights its adaptability to environmental stressors and reinforces its role in maintaining biological functions, particularly in modulating skin health [46,51].

Linoleic acid, an omega-6 polyunsaturated fatty acid, is essential for maintaining skin barrier integrity by facilitating ceramide synthesis, enhancing hydration, and reducing transepidermal water loss. Its anti-inflammatory properties include modulating cytokine production and inhibiting arachidonic acid metabolism, effectively soothing sensitive or irritated skin. Additionally, linoleic acid regulates sebum production and combats comedogenesis, addressing acne and textural irregularities, thus contributing significantly to overall skin health and appearance. Furthermore, linoleic acid has been reported to exhibit anti-tyrosinase activity, making it a valuable compound for reducing hyperpigmentation and promoting a more even skin tone, further enhancing its role in cosmeceutical applications [52,53,54,55].

In contrast, monounsaturated fatty acids like oleic or palmitoleic acid offer unique benefits in skincare formulations. These MUFAs have significant emollient properties, which make them excellent for providing hydration without blocking pores, being suitable for people with oily or combination skin. Furthermore, their reported antioxidant properties can help to protect against environmental damage, preventing premature aging and, consequently, promoting a more youthful appearance [56,57].

Diterpenes, represented by compounds such as neophytadiene (52.71 mg/100 g d.w) and phytol (24.87 mg/100 g d.w), might also contribute to the antioxidant and antiaging properties of the CP1 extract. Neophytadiene has presented potent antioxidant activity, thereby protecting the skin from premature aging and photoaging [58]. Furthermore, it also possesses anti-inflammatory properties [58]. Additionally, phytol and its derivatives exhibit collagen synthesis-stimulating effects, promoting skin elasticity and firmness, essential attributes for maintaining a youthful skin appearance [59]. There are also reports of anti-inflammatory activity, which indicate the potential to treat skin irritation [60], and it is already used as a natural fragrance, adding sensory appeal to cosmetic products [61]. A recent study also reported its moderate activity against tyrosinase [62].

The identification of β-sitosterol (16.96 mg/100 g d.w), a plant sterol, in the CP1 extract further expands its cosmeceutical potential. β-Sitosterol possesses anti-inflammatory properties and modulates immune responses, making it a valuable ingredient in skincare products aimed at soothing irritated and sensitive skin. Additionally, this compound may inhibit the enzyme 5-a-reductase, thereby reducing the conversion of testosterone to dihydrotestosterone (DHT) and mitigating the effects of androgen-induced skin disorders, such as acne and hirsutism [63,64].

The analysis of the mass spectra of the most abundant compound, obtained with a retention time of 36.9 min, showed a base peak at *m*/*z* 149, characteristic of the phthalate structure, that corresponds to the [C_8_H_4_O_3_]^+^ ion. Furthermore, it also presented a peak at *m*/*z* 279, which according to the literature, corresponds to the mono phthalate fragment [C_16_H_21_O_4_]^+^ formed by the loss of an alkyl chain with eight carbons [65]. The molecular ion peak was found to have a very low intensity at *m*/*z* 390, which points to a molecular formula of C_24_H_38_O_4_, indicating that the phthalate found on CP1 can correspond to either di-(2-ethylhexyl) phthalate, di-*n*-octyl phthalate, or diisooctyl phthalate. Preparative chromatography and further characterization by NMR spectroscopy led to the identification of the compound as di-(2-ethylhexyl) phthalate (DEHP), as described in Section 3.6.2.

Phthalates are compounds utilized both as plasticizers and non-plasticizers, serving to enhance various characteristics of different products. They contribute to improving attributes such as durability, resistance, extensibility, and flexibility in plastic polymers, while also enhancing persistence in perfumes and other fragrances. The substantial prevalence of plastic pollution has resulted in the considerable accumulation of phthalates in aquatic environments [66]. Di-(2-ethylhexyl) phthalate is one of the most used phthalates as an additive in polymeric products to make them flexible. It is used in products as diverse as medical devices, packaged food, cosmetic products (to carry fragrances), and toys. It does not evaporate easily and is found in air, water, and soil [67].

In the present work, the probability of accidental contamination of the algal material during collection and/or processing is very low. In fact, all labware and solvents used were phthalate free. To corroborate the low probability of laboratory contamination, new extracts were prepared with the same macroalgae material under identical conditions but with freshly sourced solvent and thoroughly cleaned glass labware. Additionally, other samples processed in the same laboratory under identical conditions, namely two terrestrial plants collected in the Azores, *Laurus azorica* and *Hedychium gardnerianum* (data not published), and using the same solvents and materials, consistently showed no trace of phthalates, further supporting the robustness of our methodology. The subsequent GC-MS analysis mirrored the initial findings, indicating similar levels of phthalate compounds as previously observed. A second new extract was prepared with the same conditions but using a new batch of macroalgae that was collected a year later from the exact same coordinates. By repeating the process at the same location and under the same conditions but at a different time point, the aim was to assess whether the presence of the phthalate compound was a recurring situation or an isolated event. The analysis showed once again its presence, which is strong evidence that the phthalate is a macroalgae component.

It is difficult to definitively determine the real origin of this compound. A recent review about the origin of phthalates on algae found that DEHP has been identified in many species across the three classes of macroalgae, including *C. racemosa* [68]. DEHP was found to be biosynthesized by some of the species from where it was isolated, while in others its origin was unclear or attributed to bioaccumulation [68].

Nevertheless, considering what was referenced in the previous paragraphs, it is more likely that this compound results from some interaction between the algae and the environment, including bioaccumulation and/or biosynthesis.

Phthalate concentrations in marine environments and seaweed vary widely due to environmental factors such as industrial discharges, urban runoff, and plastic pollution. Reported concentrations in seawater range from nanograms per liter to micrograms per liter [69,70,71], while in seaweed biomass, levels are typically in the nanogram to microgram per gram range [72,73,74]. The levels detected in this study were comparatively higher, which may be explained by local environmental contamination, differences in analytical and extraction methods, or the unique biological machinery of *C. prolifera*. Each seaweed species has distinct metabolic pathways and bioaccumulation mechanisms, which can influence the uptake, storage, and transformation of contaminants like phthalates.

The presence of DEHP as the major compound in the CP1 extract may explain the low antioxidant activity of the extract, despite the presence of other antioxidant agents such as neophytadiene, since DEHP has been reported in the literature to exert pro-oxidant effects [52].

The GC-MS analysis of the CP1 extract described in the previous paragraphs provided valuable insights into its chemical composition. The identified compounds, including saturated and unsaturated fatty acids, diterpenes, and sterols, exhibit a diverse range of bioactive properties related to the aging process, and show the potential of *C. prolifera* as a source of ingredients (enriched fractions or pure compounds) with cosmeceutical applications. However, the presence of DEHP in such high amounts might be problematic considering that this phthalate is reported to possess endocrine, testicular, ovarian, neural, hepatotoxic, and cardiotoxic effects [75]. This could be a critical challenge that must be addressed to enable the safe and regulated use of this seaweed species in industry. This question will be further examined in the following sections.

#### 2.2.3. Extracts Fractionation

Considering that the results of the previous section do not allow for a clear identification of the compounds responsible for the activity exhibited by extract CP1, it was fractionated using liquid–liquid extraction. This process resulted in three distinct fractions: CP1.1 (hexane fraction: 898.1 mg), CP1.2 (ethyl acetate fraction: 358.1 mg), and CP1.3 (aqueous fraction: 410.0 mg). These fractions were tested for their antioxidant and antiaging activities, and it was found that CP1.2 showcased the most robust antioxidant activity among the fractions with an EC_50_ value of 116.21 ± 0.41 µg/mL on the ABTS assay, indicating enhanced potency in comparison to CP1 (EC_50_ higher than 250 µg/mL). In the opposite sense, CP1.1 and CP1.3 displayed minimal antioxidant activity, lower than that presented by the original extract, meaning that the components of the extract responsible for its antioxidant effects were concentrated on the ethyl acetate fraction (CP1.2).

Moving to enzyme inhibition assays, CP1 demonstrated an interesting anti-tyrosinase activity, with an IC_50_ of 31.3 ± 0.37 µg/mL, while CP1.2 also showed slightly lower tyrosinase inhibition (40.8 ± 0.21 µg/mL). Fractions CP1.1 and CP1.3 were not active against this enzyme, showing again that most of the bioactive compounds were retained in the fraction of intermediate polarity (CP1.2). While CP1 exhibited moderate inhibition of elastase, with a percentage of inhibition of 33.5 ± 0.21% at 250 µg/mL, the fractions CP1.1, CP1.2, and CP1.3 displayed minimal to negligible effects. The same was observed for the inhibition of collagenase, since the fractions were less active than the extract CP1, which is already a low-active extract against this enzyme.

The observation of these results showed that the only fraction worthy of further purification was CP1.2, the ethyl acetate fraction of CP1. Thus, the CP1.2 was fractionated by Sephadex LH-20 column chromatography, since the analytical TLC analysis showed an irreversible affinity of some extract components towards silica gel 60. The CP1.2 fraction originated 12 sub-fractions, named CP1.2.1 to CP1.2.12, as shown in Table S4 (Supplementary Material). In the effort to identify the compounds responsible for the activities observed, the subfractions obtained underwent testing in identical assays as the raw extract and initial fractions, as outlined in Table 2 and Table 3 for antioxidant and antiaging activities, respectively.

Regrettably, the antioxidant activity level previously noted in CP1.2 was lost and seemed to spread across its subfractions, as evidenced by the overall diminished activity levels found for these samples.

The decrease in activity seen when comparing CP1.2 with its subfractions (CP1.2.1 to CP1.2.12), which mirrors earlier observations when comparing CP1 and its fractions in some of the activity assays, suggests that the activity detected in the more complex samples might stem from a synergistic interplay among certain components. This synergistic effect appears to dissipate upon fractionation, probably because the compounds exerting the activity were split into different fractions. Indeed, the phenomenon of diminished activity post-fractionation is a well-documented occurrence in the field of natural products, often leading to unsuccessful isolation attempts with botanical extracts [76].

Similarly, the results for tyrosinase inhibition (Table 3) showed lower potency in the subfractions (0 to 39.4% at 250 µg/mL) compared to CP1 and CP1.2 (86.4 and 80.7% at the same tested concentration). In fact, the subfractions with better activity were CP1.2.10 and CP1.2.11, and their activity at the screening concentration (39.0 and 39.4% at 250 µg/mL) was nearly 2-fold lower when compared to CP1 and CP1.2.

In contrast to the findings in the antioxidant and tyrosinase inhibition assays, subfraction CP1.2.5 exhibited markedly higher activity in elastase and collagenase inhibition compared to both the corresponding fraction (CP1.2) and the original extract (CP1). For elastase, this sample displayed an IC_50_ of 45.9 ± 0.75 µg/mL, highlighting its remarkable efficacy in this assay. As mentioned earlier, this is the first report of this activity in the *Caulerpa* genus, and the level of activity is, as far as it was possible to assess, the highest ever found on green macroalgae.

In terms of collagenase activity, subfraction CP1.2.5 demonstrated an IC_50_ of 90.6 ± 0.21 µg/mL (Table 3), which was 1.77 times higher than that of EDTA, the standard compound used as a positive control. However, unlike EDTA, which exerts its inhibition by chelating the metallic cofactor essential for collagenase function, CP1.2.5 did not display any chelating activity (EC_50_ > 250 µg/mL. This discrepancy suggests a different mode of inhibition, wherein the components of CP1.2.5 likely interact directly with the enzyme, thereby impeding its activity. This distinct mechanism of action highlights the potential of CP1.2.5 constituents as novel inhibitors of collagenase.

In this case, where the subfraction presented a great level of activity on assays while the precursor extract/fraction had low or no activity at all, two different factors can come into action. The first one is simply a change in the relative proportion of the components within the sample—by removing some components, the relative proportion of a specific compound increases, and its effect becomes more preponderant on the activity presented by the fraction [76]. The second factor, although less probable, involves antagonistic effects, i.e., the possibility that certain components within the subfraction may counteract or mitigate the activity of others. This phenomenon could arise due to complex interactions between individual compounds, where one component might suppress or nullify the activity of another. Such antagonistic effects can sometimes mask the true potential of a sample when assessed as a whole extract or fraction but become more apparent upon fractionation when individual components are isolated [76].

The chemical analysis of subfraction CP1.2.8 showed that this comprises solely di-(2-ethylhexyl) phthalate, the phthalate characterized and identified as the main constituent of the CP1 extract. Despite being a prominent component of the extract (see Figure 2), the low activity in all assays of the CP1.2.8 subfraction demonstrated the inactivity of this phthalate as an antioxidant and ECM-degrading enzyme inhibitor. In fact, DEHP is reported to exert pro-oxidant effects [77] and to enhance the activity of matrix metalloproteinases [78] in vivo.

**Figure 4:**
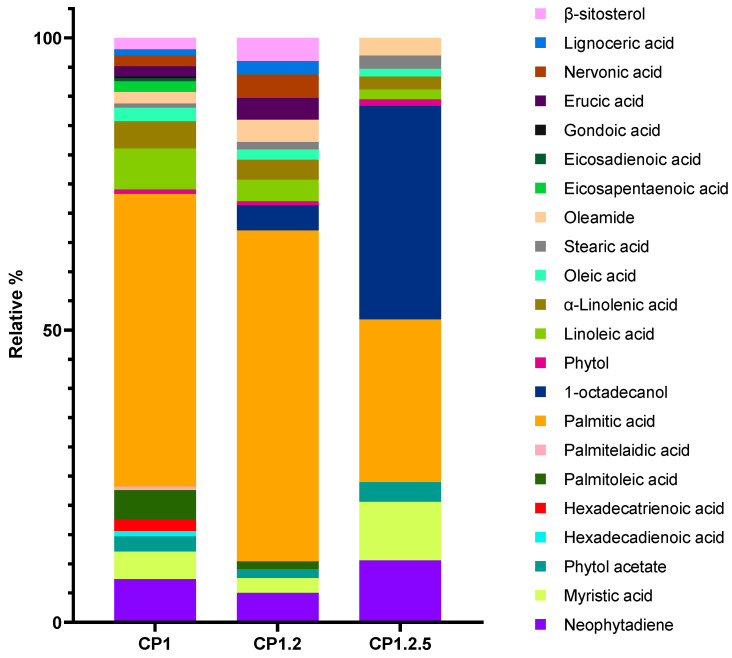
Comparison of the GC-MS profiles of CP1, CP1.2, and CP1.2.5. Corrected relative % excluding DEHP.

To gain a deeper insight into the changes in chemical profile potentially associated with the activities exhibited by CP1.2 and CP1.2.5, as well as the disparities observed compared to CP1, the composition of these fractions was assessed by GC-MS. One of the most significant changes was the expressive and progressive reduction in the proportion of the phthalate and a decreasing complexity in the chemical composition of each sample. The reduction in phthalate content in these two fractionation steps suggests that it is feasible to use this procedure as a way of reducing the risk of phthalate presence. The comparison of the relative % of each compound found on the GC-MS profile of the three samples is presented in Figure 4. Considering that the identified phthalate is inactive for the bioactivities tested (Table 2 and Table 3), and to evaluate the most significant variations of the less abundant compounds, the relative proportion was corrected to include only the remaining constituents.

A comparison between CP1 and CP1.2 revealed a significant disappearance of most unsaturated fatty acids and a decrease in the proportion of remaining ones except for erucic and nervonic acids. Nonetheless, there is not a significant decrease in the inhibitory activity against tyrosinase between CP1.2 and CP1, despite unsaturated fatty acids like oleic acid, linoleic acid, and α-linolenic acid have been reported to inhibit tyrosinase activity [43,44]. The reduction in the proportion of the phthalate observed for CP1.2 (from 37.9% in CP1 to 13.4% in CP1.2) might have contributed to this activity, since the decrease in the proportion of a major compound, which was inactive, can lead to an increase in the proportion of constituents that might be active. Palmitic acid, which has been reported to enhance tyrosinase activity [43], remained as one of the predominant compounds in both CP1 and CP1.2, constituting more than 50% of the compounds considered. The activity exhibited by the two samples showed that the effect of palmitic acid may be significantly nullified by the inhibitory effect of the unsaturated fatty acids mentioned above.

In fraction CP1.2, the doubling of oleamide, erucic acid, nervonic acid, lignoceric acid, and β-sitosterol proportions was, most likely, related to a two-fold increase in ABTS assay activity compared to CP1 (Table 3), since all these compounds are reported in the literature as possessing antioxidant properties [79,80,81,82]. In CP1.2.5, the proportion of β-sitosterol, erucic acid, nervonic acid, and lignoceric acid was decreased, coinciding with a significant decrease in the antioxidant activity, which further emphasizes the potential role of those compounds in the observed antioxidant activities.

The appearance of 1-octadecanol in small proportions on fraction CP1.2, which was not detected in CP1, is likely due to trace amounts concentrated during fractionation. The proportion of this compound was more pronounced in subfraction CP1.2.5. This fraction was less complex and exhibited expressive variations in the relative proportions of compounds, potentially explaining its distinct activities compared to CP1 and CP1.2.

The enhanced relative amount of 1-octadecanol was concomitant with improved inhibitory activity against elastase and collagenase. There is no sufficient scientific support to attribute this increased inhibitory effect solely to 1-octadecanol. To the best of our knowledge, the activity of this metabolite against these enzymes has not been reported in the literature. This increase may have been related to synergistic effects between other constituents present in the sample. In fact, studies about the effect of the constituents identified in CP1.2.5 on the activity of elastase and collagenase enzymes are practically non-existent. The presence of such a significant proportion of 1-octadecanol in this subfraction implies a greater potential for its application in cosmetic formulations, since 1-octadecanol is widely used in cosmetics and personal care products mainly due to its emollient power, ability to improve the stability and bioavailability of active agents, and its human and environmental safety profile [83,84]. The Cosmetic Ingredient Review and other regulatory agencies consider 1-octadecanol safe for use in cosmetics due to its low oral and dermal toxicity [85].

The contributions of neophytadiene, myristic acid, and phytol acetate should also be considered responsible for the increase in the activity against elastase and collagenase, as their proportions were also substantially increased in CP1.2.5. In fact, the variations in the collagenase and elastase inhibitory activity between CP1, CP1.2, and CP1.2.5 were concomitant with the variation in the proportion of these compounds, especially neophytadiene and myristic acid.

## 3. Materials and Methods

### 3.1. General

The ^1^H- and ^13^C-NMR, HSQC, HMBC, DEPT, COSY, and H2BC spectra were measured on Bruker Avance 300 (300.13 MHz for ^1^H and 75.47 MHz for ^13^C), using tetramethylsilane (TMS) as the internal standard. Chemical shifts were reported in δ units (ppm) and coupling constants (*J*) in Hz. The MS spectra were obtained using ESI(+) with a Q-Tof_2_ mass spectrometer (Manchester, UK).

Analytical thin-layer chromatography (TLC) was performed in silica gel 60 (Merck F_245_ plates), and the products were visualized with an ultraviolet lamp (254 and/or 365 nm).

### 3.2. Standards and Reagents

*N*,*O*-bis(trimethylsilyl) trifluoroacetamide (BSTFA) (99% purity); trimethylchlorosilane (TMSCl) (99%); myristic, linoleic, and oleic acids, (≥99%); β-sitosterol (97%); squalene (≥98%); phytol (≥97%); dibutyl phthalate (99%); oleamide (≥98.5%); heptadecanoic methyl ester (≥98%); octadecane (99%); 1,1-diphenyl-2-picryl-hydrazyl (DPPH); 6-hydroxy-2,5,7,8-tetramethylchroman-2-carboxylic acid (Trolox); diammonium 2,2′-azino-bis(3-ethylbenzothiazoline-6-sulfonate) (ABTS); potassium persulfate; tyrosinase; L-tyrosine; kojic acid; monosodium phosphate; sodium phosphate dibasic; elastase; N-methoxysuccinyl-Ala-Ala-Pro-Val-p-nitroanilide; N-methoxysuccinyl-Ala-Ala-Pro-chloromethylketone; 2-[4-(2-hydroxyethyl)piperazin-1-yl]ethanesulfonic acid (HEPES); N-[3-(2-Furyl)acryloyl]-Leu-Gly-Pro-Ala (FALGPA); N-[Tris(hydroxymethyl)methyl]-2-aminoethanesulfonic acid (TES) sodium salt; ninhydrin; citric acid; sodium citrate; EDTA; hyaluronidase; hyaluronic acid; calcium chloride; sodium aurothiomalate; sodium acetate; acetic acid and hydrochloric acid; dimethylsulfoxide (DMSO); dichloromethane; chloroform; and ethyl acetate were supplied by Sigma-Aldrich. Pyridine was from Fisher Scientific. Collagenase was supplied by Merck. Tin (II) chloride was obtained from Riedel-deHaën. Methanol was purchased from Honeywell. All solvents used were HPLC analytical grade and phthalate-free.

### 3.3. Macroalgae Collection

Three kilograms of *Caulerpa prolifera* (Forsskål) J.V. Lamouroux species were collected on a rockpool located in Mosteiros (in the area centered around these coordinates: 37°53′57′′ N, 25°49′18′′ W), western coast of São Miguel Island, Azores, in July 2020. The algae were collected and transported using metal utensils and 100% cotton bags, respectively. One kg of *C. prolifera* was collected in the same location and using the same methodology, one year later, to evaluate the presence of phthalate.

An intact specimen was delivered to the Department of Biology of the University of Azores for specialized identification. A voucher was created and was further deposited at the Ruy Telles Palhinha Herbarium (AZB) under the code SMG-20-17.

Following collection, the fresh mass of macroalgae underwent cleaning to remove sand, small rocks, and epiphytes. Subsequently, it was thoroughly washed with deionized water to eliminate excess salts. The material was then cut into about 1 cm long pieces and dried in darkness at room temperature with a dehumidifier. Once all the material was dehydrated, it was ground until a fine powder was achieved.

### 3.4. Moisture Determination

Four portions of fresh macroalgae (2.31 ± 0.05 g) were placed on petri dishes and then dried in an oven with forced ventilation at 40 °C. The mass of macroalgae in each petri dish was measured every two days until a constant weight was reached. 

The percentage of moisture was calculated as follows:

%water= 100 − [(Dry mass/Fresh mass) × 100].

The dry mass obtained was 0.35 ± 0.03 g, which represents a water content of 85.01 ± 0.99%.

### 3.5. Extraction

The dried biomass of *C. prolifera* (200 g) was extracted by maceration sequentially with dichloromethane, acetone, and ethanol (96%) to create extracts CP-1, CP-2, and CP-3, respectively. For each extract, the mass of vegetal material/volume of solvent ratio was 1:10, and the solvent was renewed 3 times every 72 h to ensure a better extraction yield. To minimize the risks of degradation during the extraction, it took place at room temperature (18–20 °C), in the darkness, and in a closed system with minimal oxygen exposure, until the extraction vessels were filled to capacity. The extract was then filtered using a glass filtration unit with sintered plate and concentrated with the rotavapor. All sample processing was carried out in total absence of plastic materials. Only glass labware, cotton bags, and metalware were in contact with both the alga and prepared extracts. The dichloromethane extract from the second *C. prolifera* collection was obtained using the same extraction methodology as described previously.

### 3.6. Fractionation

#### 3.6.1. Liquid–Liquid Partition

Extract CP1 (1.81 g) was dissolved in 600 mL of deionized water, and then a liquid–liquid partition was performed with chloroform. The chloroform fraction was collected and concentrated at the rotavapor. Subsequently, the aqueous phase was extracted with ethyl acetate, which was then collected and concentrated. The aqueous phase was then lyophilized to eliminate the water. Three fractions of the CP1 extract with different polarities were thus obtained: CP1.1 (chloroform fraction—898.1 mg); CP1.2 (ethyl acetate fraction—358.1 mg); CP1.3 (aqueous fraction—410.0 mg).

#### 3.6.2. Column Chromatography

Fraction CP1.2 (0.36 g) was dissolved with the aid of ultrasound in 4 mL of CHCl_3_:methanol (1:1) and then filtered in a Whatman 3 filter. The Sephadex LH-20 Column was packed as follows: the exit of the chromatographic column (30 cm length × 1 cm internal diameter) was plugged with cotton to retain the solids, and a small layer of sand was placed upon it. Sephadex LH-20 (25 g) was swollen with methanol overnight. The swollen Sephadex LH-20 slurry was poured into the column in a continuous motion. The column was rinsed with methanol. Before applying the sample, the column was equilibrated with 300 mL of CHCl_3_:methanol (1:1). The filtered CP1.2 fraction was loaded onto the column, and the elution was run with CHCl_3_:methanol (1:1) at a constant flow rate. When all the sample appeared to have exited the column, the eluent was changed into methanol. Twelve different fractions (CP1.2.1 to CP1.2.12) were obtained, according to the TLC results.

Fraction CP1.2.8.

The analytical TLC of this fraction showed that it was composed of only one compound, so it was characterized by NMR for identification.

Di-(2-ethylhexyl)phthalate: ^1^H NMR (300.13 MHz, CDCl_3_, TMS): δ 7.68–7.74 (2H, dd, *J* = 6.9 Hz and 3.63 Hz, H-3, H-6); 7.56–7.50 (2H, dd, *J* = 6.9 Hz and 3.63 Hz, H-4, H-5); 4.27–4.17 (4H, m, H-1′, H1′′); 1.72–1.65 (2H, m, H-2′, H-2′′); 1.44–1.39 (4H, m, H-7′, H-7′′); 1.33 (4H, m, H-5′, H5′′); 1.25 (8H, m, H-3′, H3′’, H-4′, H-4′′); 0.92 (6H, t, H-8′, H-8′′); 0.89 (6H, t, H-6′, H-6′′) ppm.

^13^C NMR (75.47 MHz, CDCl_3_, TMS): δ 167.8 (C=O); 132.4 (C-1, C-2); 130.9 (C4, C5); 128.8 (C-3, C6); 68.2 (C-1′, C-1′′); 38.7 (C-2′, C-2′′); 30.4 (C-3′, C-3′′); 28.9 (C-4′, C-4′′); 23.7 (C-7′, C-7′′); 23.0 (C-5′, C-5′′); 14.1 (C-6′, C-6′′); 11.0 (C-8′, C-8′′) ppm.

ESI-MS: *m/z* 391 [M + H]^+^, *m/z* 413 [M + Na]^+^, *m/z* 803 [2M + Na]^+^

### 3.7. GC-MS Analysis

#### 3.7.1. Derivatization Procedure

To increase the volatility of the compounds in the extract and thus the sensibility of the GC-MS analysis, nearly 20 mg of dried dichloromethane extract from *C. prolifera* (CP1) was dissolved in 1 mL of dichloromethane. This mixture was silylated by adding 250 μL of pyridine, 250 μL of BSTFA, and 50 μL of TMSCl, according to Seca et al. [86]. The mixture was maintained at 70 °C for 30 min and then immediately injected into the GC-MS. The quantity of silylation reagents (BSTFA and TMSCl) used was sufficient to ensure the silylation of all hydroxy groups present in the compounds, including those present in the carboxylic group.

#### 3.7.2. GC-MS Experimental Conditions

GC-MS analyses were performed using a GC-MS QP2010 Ultra Shimadzu. The separation of compounds was carried out in a DB-5 J&W capillary column (30 m × 0.25 mm inner diameter, 0.25 μm film thickness) using helium as the carrier gas (1.13 mL/min). The chromatographic conditions were as follows: initial temperature, 100 °C for 2 min; temperature rate of 10 °C/min up to 150 °C, at 3 °C/min up to 235 °C, at 10 °C/min up to 260 °C, at 2.3 °C/min up to 300 °C which was maintained for 5 min; injector temperature 320 °C; transfer-line temperature, 300 °C; and split ratio, 1:50. The mass spectrometer was operated in the electron impact (EI) mode with an energy of 70 eV, and data were collected at a rate of 1 scan/s over a range of *m*/*z* 50–1000. The ion source was kept at 200 °C. The peaks from the total ion chromatogram were identified by comparing their mass spectra with the equipment mass spectral library (NIST 14 Mass Spectral and Wiley Registry of Mass Spectral Data).

For quantification purposes, three independent replicates of each sample were submitted for the derivatization procedure, and each one was analyzed by GC-MS. The internal standard method was applied, and the amount of each metabolite present in each of the replicates was obtained from the calibration curves obtained by injection of known concentration solutions of each standard (or its TMS derivatives in case of the presence of hydroxy groups), All the injected samples and standards solutions contained a fixed quantity of internal standard (octadecane). Values of correlation coefficients confirmed linearity of the calibration plots. The concentrations of the standards were chosen to guarantee the quantification of each compound in the samples by interpolation in the calibration curve. The results were expressed in mg of compound per 100 g of dry weight of algae as the mean (±standard deviation) of the three replicates.

### 3.8. Biological Activities

A stock solution of each sample was prepared at 50 mg/mL in DMSO, from which the test samples were then prepared by diluting in the appropriate test medium. In microplate assays, absorbance values were measured using a BioRad Microplate Reader Model 680 (Bio-Rad Laboratories, Inc., Hercules, CA, USA) at the wavelengths referred to below for each test.

#### 3.8.1. DPPH Radical Scavenging Activity

Antioxidant activity was assayed by the 1,1-diphenyl-2-picryl-hydrazyl (DPPH) radical scavenging assay [87]. Serial dilutions of the studied extracts or reference compound (Trolox) were carried out in 96-well microplates using concentrations of 250, 125, 62.5, 31.25, 15.63, 7.81, 3.91, 1.95, 0.977, 0.488, and 0.244 μg/mL in methanol. DPPH dissolved in methanol was added to the microwells, yielding a final concentration of 45 µg/mL, and the absorbance at 515 nm was measured after 30 min in the dark. In each assay, a control was prepared, in which the same amount of solvent was substituted for the sample or standard. The percentage of antioxidant activity (% AA) was calculated as:

%AA = [(Abs_control_ − Abs_sample_)/Abs_control_] × 100 (1)

where Abs_control_ is the absorbance of the control and Abs_sample_ is the absorbance of the alga extract or standard. All assays were carried out in triplicate and the results expressed as IC_50_, i.e., as the concentration yielding 50% scavenging of DPPH, calculated by interpolation from the % AA vs. concentration curve.

#### 3.8.2. ABTS Radical Scavenging Assay

The method of Re et al. [88] was adopted to perform the ABTS radical scavenging assay. The stock solutions included a 7 mM ABTS solution (2,2′-azinobis-(3 ethylbenzothiazoline 6 sulfonic acid)) and a 2.4 mM potassium persulfate solution. The working solution was prepared by mixing the two stock solutions in equal quantities and allowing them to react for 12–16 h at room temperature in the dark. The solution was then diluted by mixing 1 mL ABTS solution with the amount of methanol necessary to obtain an absorbance of 0.7 at 734 nm. Serial dilutions of the studied extracts or reference compound (Trolox) were carried out in 96-well microplates using concentrations of 250, 125, 62.5, 31.25, 15.63, 7.81, 3.91, 1.95, 0.977, 0.488, and 0.244 μg/mL in methanol. The ABTS solution was then added to the microwells, and after 8 min of incubation, the absorbance was recorded at 750 nm. In each assay, a negative control was prepared, in which the same amount of solvent substituted the sample. The percentage of antioxidant activity (% AA) was calculated as:

%AA = [(Abs_control_ − Abs_sample_)/Abs_control_] × 100 (2)

where Abs_control_ is the absorbance of the ABTS radical + methanol and Abs_sample_ is the absorbance of the ABTS radical + the sample/standard.

All assays were carried out in triplicate, and the results are expressed as IC_50_, i.e., as the concentration yielding 50% scavenging of ABTS, calculated by interpolation from the% AA vs. concentration curve.

#### 3.8.3. Ferrous Chelating Activity

The Fe^2+^ chelating ability of the extracts was measured by the ferrous iron-ferrozine complex method [89]. Briefly, the reaction mixture containing 2 mM FeCl_2_ (10 µL), 5 mM ferrozine (10 µL), and 100 µL of serial concentrations of extracts or fractions (ranging between 0.244 µg/mL and 250 µg/mL in methanol) were mixed in a 96-well plate and incubated for 10 min at 27 °C. The absorbance was at 550 nm. The absorbance of the control was determined by replacing the extract with methanol. EDTA (0.195–100 µg/mL) was used as a positive control. The ability of the sample to chelate ferrous ion was calculated:

Chelating effect (%) = [(Abs_control_ − Abs_sample_)/Abs_control_] × 100 (3)

where Abs_control_ is the absorbance of the ferrozine-ferrous iron complex + methanol and Abs_sample_ is the absorbance ferrozine-ferrous iron complex + sample/standard. 

All assays were carried out in triplicate, and the results are expressed as IC_50_, i.e., value at which the concentration of the sample chelated 50% of the ferrous iron, calculated by interpolation from the % chelating effect vs. concentration curve.

#### 3.8.4. Hyaluronidase Inhibition Assay

This assay was conducted following the Sigma protocol with minor adjustments [90]. In a microplate, 25 µL of 5 U/mL of hyaluronidase prepared in a 20 mM sodium phosphate buffer (pH 7.0) with 77 mM sodium chloride and 0.01% BSA was pre-incubated with 50 µL of the sample in concentrations of 250, 125, 62.5, 31.25, 15.63, 7.81, 3.91, 1.95, 0.977, 0.488, and 0.244 μg/mL for 10 min at 37 °C. Subsequently, the assay was initiated by adding 25 µL hyaluronic acid (0.015% in 300 mM sodium phosphate, pH 5.35) to the incubation mixture and incubated for an additional 30 min at 37 °C. Undigested hyaluronic acid was precipitated with a 200 µL acid albumin solution composed of 0.1% bovine serum albumin in 24 mM sodium acetate and 79 mM acetic acid (pH 3.75). After standing at room temperature for 10 min, the absorbance of the reaction mixture was measured at 600 nm. The absorbance in the absence of the enzyme (blank) served as the reference value for maximum absorbance. Enzyme activity was verified by a control experiment conducted simultaneously, in which the enzyme was pre-incubated with a buffer instead, followed by the assay procedures described above. The inhibitory activity of the test compound was determined as follows:

%Inhibition = [1 − (Abs_blank_ − Abs_sample_)/(Abs_blank_ − Abs_control_)] × 100 (4)

where Abs_blank_ is the absorbance of the reaction without the enzyme, Abs_sample_ is the absorbance of the extract or standard compound sodium aurothiomalate, and Abs_control_ is the absorbance of the enzyme without the inhibitor. The IC_50_ value, which was the sample concentration that inhibited 50% of the enzyme activity, was determined by interpolation from the % hyaluronidase inhibition vs. concentration curve.

#### 3.8.5. Tyrosinase Inhibition Assay

The extracts were assayed by adapting the tyrosinase inhibition method described by Shimizu et al. [91] and modified by Manosroi et al. [92]. In brief, 25 μL of a tyrosinase enzyme solution (135 U/mL), 25 μL of ten serial concentrations of the extracts (0.488 μg/mL to 250 μg/mL dissolved in 100 mM phosphate buffer, pH 6.8 containing no more than 2.5% DMSO), and 100 μL of a phosphate buffer were mixed in a 96-well plate and incubated at 37 °C for 20 min. Then, 50 μL of 1.66 mM of a tyrosine solution in 100 mM of a phosphate buffer, pH 6.8, was added. The enzyme activity was measured at 490 nm every 10 min for 30 min. Kojic acid at 0.293–150 μg/mL was used as a positive control. The experiments were carried out in triplicate. For each concentration, enzyme activity was calculated as a percentage of the velocities compared to that of the assay using a buffer without any inhibitor. The IC_50_ value, which was the sample concentration that inhibited 50% of the enzyme activity, was determined by interpolation from the % tyrosinase inhibition vs. concentration curve.

#### 3.8.6. Elastase Inhibition Assay

The extracts were assayed by the method described by Ndlovu et al. [93] with some modifications. In brief, 25 μL of the elastase enzyme solution (0.3 U/mL), 50 μL of ten serial concentrations of the extracts or fractions (0.488 μg/mL to 250 μg/mL dissolved in 100 mM HEPES buffer, pH 7.5 containing no more than 2.5% DMSO), and 125 μL of the HEPES buffer were mixed in a 96-well plate and incubated at room temperature for 20 min. Then, 50 μL of *N*-methoxysuccinyl-Ala-Ala-Pro-Val-p-nitroanilide (1 mM) was added. The enzyme activity was measured at 405 nm at the moment of substrate addition and after 40 min of incubation at 25 °C. *N*-Methoxysuccinyl-Ala-Ala-Pro-chloromethyl ketone at 0.019–20 μg/mL was used as a positive control. The experiments were carried out in triplicate. For each concentration, enzyme activity was calculated as a percentage of the velocities compared to that of the assay using a buffer without any inhibitor. The IC_50_ value, which was the sample concentration that inhibited 50% of the enzyme activity, was determined by interpolation from the % elastase inhibition vs. concentration curve.

#### 3.8.7. Collagenase Inhibition Assay

An adaptation of the method by Mandl et al. [94] was used to determine anti-collagenase activity. The following was added to 2 mL test tubes: 25 μL of collagenase solution (0.8 U/mL); 25 μL of a TES buffer (50 mM) with 0.36 mM of calcium chloride, pH 7.4; and 50 μL of the test sample or the reference compound EDTA (with concentrations ranging between 15.6 and 250 μg/mL). The tubes were incubated in a water bath at 37 °C for 20 min. Thereafter, 50 μL of a FALGPA (1 mM) solution was added to the tubes and further incubated for 60 min at 37 °C. To all tubes, 200 μL of a solution containing equal volumes of a 1.6 mg/mL tin chloride (II) solution in 200 mM of a citrate buffer, pH 5, and 50 mg/mL of a ninhydrin solution in DMSO was added. All tubes were placed in a water bath (100 °C) for 5 min and left to cool to room temperature before adding 200 μL of 50% isopropanol to each tube. The contents in the tubes were then transferred to respective wells in 96-well plates. Absorbance was detected at 550 nm. The percentage of collagenase inhibition was calculated as:

% Collagenase inhibition = [(Abs_control_ − Abs_sample_)]/Abs_control_] × 100 (5)

where Abs_control_ is the absorbance of the buffer + collagenase and Abs_sample_ is the absorbance of the buffer + collagenase + sample/standard.

All assays were carried out in triplicate, and the results are expressed as IC_50_, i.e., as the concentration yielding 50% of collagenase inhibition, calculated by interpolation from the % collagenase inhibition vs. concentration curve.

### 3.9. Statistical Analysis

A two-way ANOVA, followed by post-hoc HSD Tukey’s test, were used to assess the significant differences between samples in each assayed biological test using the open-source software R [95] (4.2.1 for Windows) and RStudio.

## 4. Conclusions

This work describes the phytochemical study of *C. prolifera* and the determination of its potential for cosmeceutical applications. Following this investigation, this species emerged as a promising source of enriched fractions and compounds with various interesting bioactivities. The lipophilic extract of *C. prolifera* displays significant activity against target enzymes, particularly tyrosinase. However, the overall antioxidant capacity of this species appears to be limited.

The extract primarily consists of phthalate and fatty acids, both saturated and unsaturated. While saturated fatty acids dominate in quantity, their unsaturated counterparts exhibit a more diverse and rich profile. Also, other compound classes, such as diterpenes and sterols, are present in the extract. In addition to the bioactivities observed for the extract, the known applications of the compounds identified, such as fragrance enhancing, texture, and emollient properties in cosmetic formulations, suggest considerable potential for *C. prolifera* in cosmeceutical development, not only as a bioactive agent but also as an excipient contributing to improving the physicochemical characteristics of the formulation.

However, the identification of di-(2ethylhexyl) phthalate as the main compound raises concerns due to its classification as an endocrine disruptor, associated with various adverse effects on health. The data indicate that *C. prolifera* seems to be able to either biosynthesize and/or bioaccumulate this phthalate, necessitating caution in its use for cosmeceutical formulations. Further fractionation of the extract has enabled the isolation of this compound into a subfraction which is inactive against the tested bioactivities, while other subfractions exhibit traces of phthalate and the best bioactivities level.

The fractionation of CP1 resulted in fractions with distinct activities compared to the initial extract, a common occurrence in natural product discovery. Phenomena such as synergy or antagonism among the various constituents of an extract can occur, influencing the observed activities. Fractionation alters the composition, sometimes resulting in a loss of activity, but it can also lead to increases due to the concentration of compounds that were previously present in minor quantities.

The chemical composition differences among CP1, CP1.2, and CP1.2.5 revealed by the GC-MS analysis may account for their divergent bioactivities. Subfraction CP1.2.5 exhibits reduced complexity, with increased proportions of certain compounds correlating with improved inhibitory activity against elastase and collagenase. The largest amount of 1-octadecanol in CP1.2.5 confirms an expected enrichment effect during fractionation and may probably be related to the inhibition of collagenase and elastase. Conversely, the antioxidant activity of CP1.2.5 decreases with the loss of specific compounds like β-sitosterol, erucic acid, nervonic acid, and lignoceric acid. The CP1.2.5 subfraction also shows a reduction in unsaturated fatty acids compared to CP1 and CP1.2, likely contributing to a lower ability to inhibit tyrosinase. Furthermore, the potential tyrosinase activating effect of palmitic acid (dominant in both CP1 and CP1.2) appears limited in CP1.2.5, since the palmitic acid proportion is lowest.

The phytochemical analysis enhanced our understanding of the potential effects of the various compounds present in *C. prolifera* on its bioactivities. This contributes to a more comprehensive valorization of this macroalga as a promising source of cosmeceutical ingredients.

## Figures and Tables

**Table 1:** Extract yields resulting from the sequential extraction of *C. prolifera*.

Extract	Solvent	Extract Mass (g)	Yield (g Extract/100 g d.w)
CP1	Dichloromethane	9.58	4.79
CP2	Acetone	1.06	0.53
CP3	Ethanol	2.71	1.36

**Table 2:** Antioxidant activity by DPPH and ABTS methods of the fractions of CP1 extract.

Sample	DPPH		ABTS	
	% AA *	EC_50_ µg/mL	% AA *	EC_50_ µg/mL
CP1 ^§^	15.3 ± 0.54	>250 ^a^	31.5 ± 0.11	>250 ^a^
CP1.1 ^§^	0	>250 ^a^	29.7 ± 0.34	>250 ^a^
CP1.2 ^§^	0	>250 ^a^	65.2 ± 0.14	116.21 ± 0.41 ^b^
CP1.3 ^§^	0	>250 ^a^	10.0 ± 0.33	>250 ^a^
CP1.2.1	0	>250 ^a^	0	>250 ^a^
CP1.2.2	0	>250 ^a^	26.6 ± 0.88	>250 ^a^
CP1.2.3	0	>250 ^a^	29.5 ± 0.61	>250 ^a^
CP1.2.4	0	>250 ^a^	16.5 ± 0.61	>250 ^a^
CP1.2.5	0	>250 ^a^	11.21 ± 0.11	>250 ^a^
CP1.2.6	0	>250 ^a^	0	>250 ^a^
CP1.2.7	0	>250 ^a^	5.2 ± 0.35	>250 ^a^
CP1.2.8	0	>250 ^a^	4.9 ± 0.95	>250 ^a^
CP1.2.9	1.2 ± 0.49	>250 ^a^	9.5 ± 0.40	>250 ^a^
CP1.2.10	1.9 ± 0.51	>250 ^a^	11.2 ± 0.06	>250 ^a^
CP1.2.11	0	>250 ^a^	12.3 ± 0.60	>250 ^a^
CP1.2.12	0	>250 ^a^	7.9 ± 0.95	>250 ^a^
Trolox	89.7 ± 0.50	7.3 ± 0.09 ^b^	87.1 ± 0.95	2.68 ± 0.08 ^c^

§ The antioxidant activity of these samples is repeated here to facilitate comparative analysis of the results. * % antioxidant activity at 250 µg/mL. In each EC_50_ column, different letters indicate significant differences (*p* < 0.05).

**Table 3:** ECM-degrading enzyme inhibition by the fractions of CP1 extract.

Sample	Elastase		Tyrosinase		Collagenase	
	% Inhibition *	IC_50_ µg/mL	% Inhibition *	IC_50_ µg/mL	% Inhibition *	IC_50_ µg/mL
§ CP1	33.5 ± 0.21	>250 ^a^	86.4 ± 0.38	31.3 ± 0.37 ^a^	12.3 ± 0.22	>250 ^a^
§ CP1.1	0	>250 ^a^	15.0 ± 0.65	>250 ^b^	0	>250 ^a^
§ CP1.2	0	>250 ^a^	80.7 ± 0.34	40.8 ± 0.21 ^c^	0	>250 ^a^
§ CP1.3	5.3 ± 0.77	>250 ^a^	0	>250 ^b^	0	>250 ^a^
CP1.2.1	0	>250 ^a^	0	>250 ^b^	0	>250 ^a^
CP1.2.2	0	>250 ^a^	13.3 ± 0.20	>250 ^b^	0	>250 ^a^
CP1.2.3	0	>250 ^a^	13.8 ± 0.76	>250 ^b^	0	>250 ^a^
CP1.2.4	0	>250 ^a^	18.4 ± 0.87	>250 ^b^	3.7 ± 0.80	>250 ^a^
CP1.2.5	84.2 ± 0.68	45.9 ± 0.75 ^b^	12.7 ± 0.69	>250 ^b^	78.1 ± 0.08	90.6 ± 0.21 ^b^
CP1.2.6	0	>250 ^a^	0	>250 ^b^	0	>250 ^a^
CP1.2.7	36.8 ± 0.92	>250 ^a^	12.9 ± 0.96	>250 ^b^	7.6 ± 0.22	>250 ^a^
CP1.2.8	0	>250 ^a^	16.0 ± 0.60	>250 ^b^	9.5 ± 0.63	>250 ^a^
CP1.2.9	0	>250 ^a^	29.6 ± 0.76	>250 ^b^	9.6 ± 0.18	>250 ^a^
CP1.2.10	0	>250 ^a^	39.0 ± 0.61	>250 ^b^	12.4 ± 0.59	>250 ^a^
CP1.2.11	0	>250 ^a^	39.4 ± 0.78	>250 ^b^	9.5 ± 0.45	>250 ^a^
CP1.2.12	0	>250 ^a^	24.5 ± 0.57	>250 ^b^	36.5 ± 0.34	>250 ^a^
NMAAK	95.5 ± 3.96	0.13 ± 0.002 ^c^	-	-	-	-
Kojic acid	-	-	99.2 ± 0.36	1.8 ± 0.13 ^d^	-	-
EDTA	-	-	-	-	96.3 ± 1.2	51.2 ± 0.12 ^c^

§ The inhibitory activity of these samples is repeated here to facilitate comparative analysis of the results. * % inhibitory activity at 250 µg/mL. In each IC_50_column, different letters indicate significant differences (*p* < 0.05).

## Data Availability

The data presented on this study are available in the article.

## Supplementary Materials

The following supporting information can be downloaded at: https://www.mdpi.com/article/10.3390/md23020083/s1, Figure S1. Chromatogram obtained by the GC-MS analysis of CP1, Figure S2. ^1^H NMR spectrum of fraction CP1.2.8., Figure S3. ^13^C NMR spectrum of fraction CP1.2.8 and Figure S4. Mass spectra of fraction CP1.2.8; Table S1. Antioxidant activity by DPPH and ABTS methods of *C. prolifera* extracts. Table S2. ECM degrading enzyme inhibition of *C. prolifera* extracts. Table S3. Compounds identified by GC/MS on the dichloromethane extract of *Caulerpa prolifera* (CP1). Table S4. Mass of fractions obtained from Sephadex column fractionation of CP1.2.

## References

[1] Xu H., Zheng Y.-W., Liu Q., Liu L.-P., Luo F.-L., Zhou H.-C., Isoda H., Ohkohchi N., Li Y.-M. (2018). Reactive oxygen species in skin repair, regeneration, aging, and inflammation. Reactive Oxygen Species (ROS) in Living Cells.

[2] Navarro C., Salazar J., Díaz M.P., Chacin M., Santeliz R., Vera I., D′Marco L., Parra H., Bernal M.C., Castro A. (2023). Intrinsic and environmental basis of aging: A narrative review. Heliyon.

[3] Shin J.-W., Kwon S.-H., Choi J.-Y., Na J.-I., Huh C.-H., Choi H.-R., Park K.-C. (2019). Molecular mechanisms of dermal aging and antiaging approaches. Int. J. Mol. Sci..

[4] Maldonado E., Morales-Pison S., Urbina F., Solari A. (2023). Aging hallmarks and the role of oxidative stress. Antioxidants.

[5] Papaccio F., D’arino A., Caputo S., Bellei B. (2022). Focus on the contribution of oxidative stress in skin aging. Antioxidants.

[6] Kathuria D., Sharma A., Verma M., Nayik G.A. (2024). Bioprospecting of Natural Sources for Cosmeceuticals.

[7] He X., Gao X., Guo Y., Xie W. (2024). Research progress on bioactive factors against skin aging. Int. J. Mol. Sci..

[8] Liu S., Yan W., Zhang J., Li Z., Guo Y. (2024). Copper ions amplify the oxidative stress caused by calcium overload leading to apoptosis. Biomed. Anal..

[9] Tumilaar S.G., Hardianto A., Dohi H., Kurnia D. (2024). A comprehensive review of free radicals, oxidative stress, and antioxidants: Overview, clinical applications, global perspectives, future directions, and mechanisms of antioxidant activity of flavonoid compounds. J. Chem..

[10] Papakonstantinou E., Roth M., Karakiulakis G. (2012). Hyaluronic acid: A key molecule in skin aging. Dermato-Endocrinology.

[11] Zolghadri S., Beygi M., Mohammad T.F., Alijanianzadeh M., Pillaiyar T., Garcia-Molina P., Garcia-Canovas F., Munoz-Munoz J., Saboury A.A. (2023). Targeting tyrosinase in hyperpigmentation: Current status, limitations and future promises. Biochem. Pharmacol..

[12] Goyal A., Sharma A., Kaur J., Kumari S., Garg M., Sindhu R.K., Rahman M.H., Akhtar M.F., Tagde P., Najda A. (2022). Bioactive-based cosmeceuticals: An update on emerging trends. Molecules.

[13] Chakdar H., Pabbi S. (2017). Algal pigments for human health and cosmeceuticals. Algal Green Chemistry.

[14] Thiyagarasaiyar K., Goh B.-H., Jeon Y.-J., Yow Y.-Y. (2020). Algae metabolites in cosmeceutical: An overview of current applications and challenges. Mar. Drugs.

[15] Wijesinghe W.A.J.P., Jeon Y.-J. (2011). Biological activities and potential cosmeceutical applications of bioactive components from brown seaweeds: A review. Phytochem. Rev..

[16] Ruocco N., Costantini S., Guariniello S., Costantini M. (2016). Polysaccharides from the marine environment with pharmacological, cosmeceutical and nutraceutical potential. Molecules.

[17] Sanjeewa K.K.A., Kim E.-A., Son K.-T., Jeon Y.-J. (2016). Bioactive properties and potentials cosmeceutical applications of phlorotannins isolated from brown seaweeds: A review. J. Photochem. Photobiol. B.

[18] Guiry M.D., Guiry G.M., AlgaeBase World-wide electronic publication, National University of Ireland, Galway. 22 December 2020. https://www.algaebase.org.

[19] Cacabelos E., Faria J., Martins G.M., Mir C., Parente M.I., Gabriel D., Sánchez R., Altamirano M., Costa A.C., Prud’homme van Reine W. (2019). First record of *Caulerpa prolifera* in the Azores (NE Atlantic). Bot. Mar..

[20] Smyrniotopoulos V., Abatis D., Tziveleka L.-A., Tsitsimpikou C., Roussis V., Loukis A., Vagias C. (2003). Acetylene sesquiterpenoid esters from the green alga *Caulerpa prolifera*. J. Nat. Prod..

[21] Abdel-Wahhab M.A., Ahmed H.H., Hagazi M.M. (2006). Prevention of Aflatoxin B1-initiated hepatotoxicity in rat by marine algae extracts. J. Appl. Toxicol..

[22] Costa L.S., Fidelis G.P., Cordeiro S.L., Oliveira R.M., Sabry D.A., Câmara R.B.G., Nobre L.T.D.B., Costa M.S.S.P., Almeida-Lima J., Farias E.H.C. (2010). Biological activities of sulfated polysaccharides from tropical seaweeds. Biomed. Pharmacother..

[23] Chaves Filho G.P., de Paula Oliveira R., de Medeiros S.R.B., Rocha H.A.O., Moreira S.M.G. (2020). Sulfated polysaccharides from green seaweed *Caulerpa prolifera* suppress fat accumulation. J. Appl. Phycol..

[24] Cengiz S., Cavas L., Yurdakoc K., Pohnert G. (2011). The sesquiterpene caulerpenyne from *Caulerpa* spp. is a lipoxygenase inhibitor. Mar. Biotechnol..

[25] Sureda A., Box A., Enseñat M., Alou E., Tauler P., Deudero S., Pons A. (2006). Enzymatic antioxidant response of a labrid fish *Coris julis* liver to environmental caulerpenyne. Comp. Biochem. Physiol. Part C Toxicol. Pharmacol..

[26] Cavalcante-Silva L., De Carvalho Correia A., Barbosa-Filho J., Da Silva B., De Oliveira Santos B., De Lira D., Sousa J., De Miranda G., De Andrade Cavalcante F., Alexandre-Moreira M. (2013). Spasmolytic Effect of caulerpine involves blockade of Ca^2+^ influx on guinea pig ileum. Mar. Drugs.

[27] Macedo N.R.P.V., Ribeiro M.S., Villaça R.C., Ferreira W., Pinto A.M., Teixeira V.L., Cirne-Santos C., Paixão I.C.N.P., Giongo V. (2012). Caulerpin as a potential antiviral drug against *Herpes simplex* Virus Type 1. Rev. Bras. Farmacogn..

[28] De Souza É.T., Pereira de Lira D., Cavalcanti de Queiroz A., Costa da Silva D.J., Bezerra de Aquino A., Campessato Mella E.A., Prates Lorenzo V., De Miranda G.E.C., De Araújo-Júnior J.X., De Oliveira Chaves M.C. (2009). The antinociceptive and anti-inflammatory activities of caulerpin, a bisindole alkaloid isolated from seaweeds of the genus *Caulerpa*. Mar. Drugs.

[29] Dembitsky V.M., Levitsky D.O., Gloriozova T.A., Poroikov V.V. (2006). Acetylenic aquatic anticancer agents and related compounds. Nat. Prod. Commun..

[30] de Napoli L., Fattorusso E., Magno S., Mayol L. (1981). Furocaulerpin, a new acetylenic sesquiterpenoid from the green alga *Caulerpa prolifera*. Experientia.

[31] Cheng L., Ji T., Zhang M., Fang B. (2024). Recent Advances in Squalene: Biological Activities, Sources, Extraction, and Delivery Systems. Trends Food Sci. Technol..

[32] Box A., Sureda A., Tauler P., Terrados J., Marbà N., Pons A., Deudero S. (2010). Seasonality of caulerpenyne content in native *Caulerpa prolifera* and invasive *C. taxifolia* and *C. racemosa* var. cylindracea in the Western Mediterranean sea. Bot. Mar..

[33] García-Sánchez M., Korbee N., Pérez-Ruzafa I.M., Marcos C., Domínguez B., Figueroa F.L., Pérez-Ruzafa Á. (2012). Physiological response and photoacclimation capacity of *Caulerpa prolifera* (Forsskål) J.V. Lamouroux and *Cymodocea nodosa* (Ucria) Ascherson meadows in the Mar Menor Lagoon (SE Spain). Mar. Environ. Res..

[34] Yap W.-F., Tay V., Tan S.-H., Yow Y.-Y., Chew J. (2019). Decoding antioxidant and antibacterial potentials of malaysian green seaweeds: *Caulerpa racemosa* and *Caulerpa lentillifera*. Antibiotics.

[35] Chaabani E., Rebey I.B., Mgaidi S., Wannes W.A., Hammemi M., Shili A., Ksouri R. (2023). Effect of extraction method on the phytochemical composition, antioxidant and antimicrobial activities of *Caulerpa prolifera* (Forssk.) J.V. Lamour. (*Chlorophyta*) extract. Int. J. Algae.

[36] Kurniawan R., Nurkolis F., Taslim N.A., Subali D., Surya R., Gunawan W.B., Alisaputra D., Mayulu N., Salindeho N., Kim B. (2023). Carotenoids composition of green algae *Caulerpa racemosa* and their antidiabetic, anti-obesity, antioxidant, and anti-inflammatory properties. Molecules.

[37] Sanger G., Wonggo D., Taher N., Dotulong V., Setiawan A.A., Permatasari H.K., Maulana S., Nurkolis F., Tsopmo A., Kim B. (2023). Green seaweed *Caulerpa racemosa*—Chemical constituents, cytotoxicity in breast cancer cells and molecular docking simulation. J. Agric. Food Res..

[38] Rosa G.P., Peixoto A.F., Barreto M.C., Seca A.M.L., Pinto D.C.G.A. (2022). Bio-Guided Optimization of *Cystoseira Abies-Marina* Cosmeceuticals Extraction by Advanced Technologies. Mar. Drugs.

[39] Choosuwan P., Praiboon J., Boonpisuttinant K., Klomjit A., Muangmai N., Ruangchuay R., Chirapart A. (2023). Inhibitory effects of *Caulerpa racemosa*, *Ulva intestinalis*, and *Lobophora challengeriae* on tyrosinase activity and α-MSH-induced melanogenesis in B16F10 melanoma cells. Life.

[40] Castejón N., Thorarinsdottir K.A., Einarsdóttir R., Kristbergsson K., Marteinsdóttir G. (2021). Exploring the potential of icelandic seaweeds extracts produced by aqueous pulsed electric fields-assisted extraction for cosmetic applications. Mar. Drugs.

[41] Kelm G.R., Wickett R.R. (2017). The role of fatty acids in cosmetic technology. Fatty Acids.

[42] Baker P., Huang C., Radi R., Moll S.B., Jules E., Arbiser J.L. (2023). Skin barrier function: The interplay of physical, chemical, and immunologic properties. Cells.

[43] Ando H., Watabe H., Valencia J.C., Yasumoto K.I., Furumura M., Funasaka Y., Oka M., Ichihashi M., Hearing V.J. (2004). Fatty acids regulate pigmentation via proteasomal degradation of tyrosinase: A new aspect of ubiquitin-proteasome function. J. Biol. Chem..

[44] Kose A. (2023). Chemical Composition and Tyrosinase Inhibitory Activities of Fatty Acids Obtained from Heterotrophic Microalgae, *S*. limacinum and C. cohnii. Appl. Biochem. Biotechnol..

[45] Ismail M.M., Ismail G.A., El-Sheekh M.M. (2024). Potential assessment of some micro- and macroalgal species for bioethanol and biodiesel production. Energy Sources Part A Recovery Util. Environ. Eff..

[46] Kamal M., Abdel-Raouf N., Alwutayd K., AbdElgawad H., Abdelhameed M.S., Hammouda O., Elsayed K.N.M. (2023). Seasonal changes in the biochemical composition of dominant macroalgal species along the egyptian red sea shore. Biology.

[47] Harwood J.L. (2023). Polyunsaturated fatty acids: Conversion to lipid mediators, roles in inflammatory diseases and dietary sources. Int. J. Mol. Sci..

[48] Simonetto M., Infante M., Sacco R.L., Rundek T., Della-Morte D. (2019). A novel anti-inflammatory role of omega-3 pufas in prevention and treatment of atherosclerosis and vascular cognitive impairment and dementia. Nutrients.

[49] Czumaj A., Śledziński T. (2020). Biological role of unsaturated fatty acid desaturases in health and disease. Nutrients.

[50] Makrantonaki E., Ganceviciene R., Zouboulis C.C. (2011). An update on the role of the sebaceous gland in the pathogenesis of acne. Dermato-Endocrinology.

[51] Terrados J., Lopez-Jimenez J.A. (1996). Fatty acid composition and chilling resistance in the green alga *Caulerpa prolifera* (Forrskal) Lamouroux (Chlorophyta, Caulerpales). Biochem. Mol. Biol. Int..

[52] Djuricic I., Calder P.C. (2021). Beneficial outcomes of Omega-6 and Omega-3 polyunsaturated fatty acids on human health: An update for 2021. Nutrients.

[53] Innes J.K., Calder P.C. (2018). Omega-6 fatty acids and inflammation. Prostaglandins Leukot. Essent. Fat. Acids.

[54] Lin T.-K., Zhong L., Santiago J. (2017). Anti-inflammatory and skin barrier repair effects of topical application of some plant oils. Int. J. Mol. Sci..

[55] Kovács D., Camera E., Póliska S., Cavallo A., Maiellaro M., Dull K., Gruber F., Zouboulis C.C., Szegedi A., Törőcsik D. (2023). Linoleic acid induced changes in SZ95 sebocytes—Comparison with palmitic acid and arachidonic acid. Nutrients.

[56] Latreille J., Kesse-Guyot E., Malvy D., Andreeva V., Galan P., Tschachler E., Hercberg S., Guinot C., Ezzedine K. (2012). Dietary monounsaturated fatty acids intake and risk of skin photoaging. PLoS ONE.

[57] Mittal A., Sara U., Ali A., Aqil M. (2009). Status of fatty acids as skin penetration enhancers—A review. Curr. Drug Deliv..

[58] Bhardwaj M., Sali V.K., Mani S., Vasanthi H.R. (2020). Neophytadiene from *Turbinaria Ornata* suppresses LPS-induced inflammatory response in RAW 264.7 macrophages and Sprague Dawley rats. Inflammation.

[59] DiNatale L., Idkowiak-Baldys J., Zhuang Y., Gonzalez A., Stephens T.J., Jiang L.I., Li W., Basson R., Bayat A. (2021). Novel rotational combination regimen of skin topicals improves facial photoaging: Efficacy demonstrated in double-blinded clinical trials and laboratory validation. Front. Med..

[60] Silva R.O., Sousa F.B.M., Damasceno S.R.B., Carvalho N.S., Silva V.G., Oliveira F.R.M.A., Sousa D.P., Aragão K.S., Barbosa A.L.R., Freitas R.M. (2014). Phytol, a diterpene alcohol, inhibits the inflammatory response by reducing cytokine production and oxidative stress. Fundam. Clin. Pharmacol..

[61] McGinty D., Letizia C.S., Api A.M. (2010). Fragrance material review on phytol. Food Chem. Toxicol..

[62] Rosa G.P., Seca A.M.L., Pinto D.C.G.A., Barreto M.C. (2024). New phytol derivatives with increased cosmeceutical potential. Molecules.

[63] Zhang P., Liu N., Xue M., Zhang M., Liu W., Xu C., Fan Y., Meng Y., Zhang Q., Zhou Y. (2023). Anti-inflammatory and antioxidant properties of β-sitosterol in copper sulfate-induced inflammation in zebrafish *Danio rerio*. Antioxidants.

[64] Khan Z., Nath N., Rauf A., Emran T.B., Mitra S., Islam F., Chandran D., Barua J., Khandaker M.U., Idris A.M. (2022). Multifunctional roles and pharmacological potential of β-sitosterol: Emerging evidence toward clinical applications. Chem. Biol. Interact..

[65] Yin P., Chen H., Liu X., Wang Q., Jiang Y., Pan R. (2014). Mass spectral fragmentation pathways of phthalate esters by gas chromatography–Tandem mass spectrometry. Anal. Lett..

[66] Eales J., Bethel A., Galloway T., Hopkinson P., Morrissey K., Short R.E., Garside R. (2022). Human health impacts of exposure to phthalate plasticizers: An overview of reviews. Environ. Int..

[67] Dobrzyńska M.M. (2016). Phthalates—Widespread occurrence and the effect on male gametes. Part 1. general characteristics, sources and human exposure. Rocz. Państwowego Zakładu Hig..

[68] Huang L., Zhu X., Zhou S., Cheng Z., Shi K., Zhang C., Shao H. (2021). Phthalic acid esters: Natural sources and biological activities. Toxins.

[69] Mi L., Xie Z., Xu W., Waniek J.J., Pohlmann T., Mi W. (2023). Air-sea exchange and atmospheric deposition of phthalate esters in the South China Sea. Environ. Sci. Technol..

[70] Wang M.H., Chen C.F., Albarico F.P.J.B., Tsai W.P., Chen C.W., Dong C. (2022). Concentrations of phthalate esters on indian ocean silky sharks and their long-term dietary consumption risks. Mar. Biol. Res..

[71] Zhang Q., Song J., Li X., Peng Q., Yuan H., Li N., Duan L., Ma J. (2019). Concentrations and distribution of phthalate esters in the seamount area of the tropical Western Pacific Ocean. Mar. Pollut. Bull..

[72] Pace A., Vaglica A., Maccotta A., Savoca D. (2024). The origin of phthalates in algae: Biosynthesis and environmental bioaccumulation. Environments.

[73] Chan H.W., Lau T.C., Ang P.O., Wu M., Wong P.K. (2004). Biosorption of di(2-ethylhexyl)phthalate by seaweed biomass. J. Appl. Phycol..

[74] Savoca D., Lo Coco R., Melfi R., Pace A. (2022). Uptake and photoinduced degradation of phthalic acid esters (PAEs) in *Ulva lactuca* highlight its potential application in environmental bioremediation. Environ. Sci. Pollut. Res..

[75] Rowdhwal S.S.S., Chen J. (2018). Toxic effects of di-2-ethylhexyl phthalate: An overview. BioMed Res. Int..

[76] Caesar L.K., Cech N.B. (2019). Synergy and antagonism in natural product extracts: When 1 + 1 does not equal 2. Nat. Prod. Rep..

[77] Wang Y., Wang T., Ban Y., Shen C., Shen Q., Chai X., Zhao W., Wei J. (2018). Di-(2-ethylhexyl) phthalate exposure modulates antioxidant enzyme activity and gene expression in juvenile and adult *Daphnia magna*. Arch. Environ. Contam. Toxicol..

[78] Shih M.-F., Pan K.-H., Cherng J. (2015). Possible mechanisms of di(2-ethylhexyl) phthalate-induced MMP-2 and MMP-9 expression in A7r5 rat vascular smooth muscle cells. Int. J. Mol. Sci..

[79] Reyes-Soto C.Y., Villaseca-Flores M., Ovalle-Noguez E.A., Nava-Osorio J., Galván-Arzate S., Rangel-López E., Maya-López M., Retana-Márquez S., Túnez I., Tinkov A.A. (2022). Oleamide reduces mitochondrial dysfunction and toxicity in rat cortical slices through the combined action of cannabinoid receptors activation and induction of antioxidant activity. Neurotox. Res..

[80] Chen S., Wang X., Wang X., Zheng W., He S., Song M., Wang H. (2022). The influence of syringic acid and erucic acid on the antioxidant properties of natural rubber: Experimental and molecular simulation investigations. Polymers.

[81] Goyal A., Dubey N., Verma A., Agrawal A. (2024). Erucic acid: A possible therapeutic agent for neurodegenerative diseases. Curr. Mol. Med..

[82] Umemoto H., Yasugi S., Tsuda S., Yoda M., Ishiguro T., Kaba N., Itoh T. (2021). Protective effect of nervonic acid against 6-hydroxydopamine-induced oxidative stress in PC-12 Cells. J. Oleo Sci..

[83] Environmental Protection Agency (2020). Supporting Information for Low-Priority Substance 1-Octadecanol (CASRN 112-92-5) Final Designation.

[84] Prieto-Blanco M.C., Fernández-Amado M., López-Mahía P., Muniategui-Lorenzo S., Prada-Rodríguez D. (2018). Surfactants in cosmetics. Analysis of Cosmetic Products.

[85] Ann Liebert M., Alcohol S., Alcohol O., Dodecanol O. (1985). Final report on the safety assessment of stearyl alcohol, oleyl alcohol, and octyl dodecanol. J. Am. Coll. Toxicol..

[86] Seca A.M.L., Gouveia V.L.M., Carmo Barreto M., Silva A.M.S., Pinto D.C.G.A. (2018). Comparative study by GC-MS and chemometrics on the chemical and nutritional profile of *Fucus spiralis* L. juvenile and mature life-cycle phases. J. Appl. Phycol..

[87] Blois M.S. (1958). Antioxidant determinations by the use of a stable free radical. Nature.

[88] Re R., Pellegrini N., Proteggente A., Pannala A., Yang M., Rice-Evans C. (1999). Antioxidant activity applying an improved abts radical cation decolorization assay. Free Radic. Biol. Med..

[89] Decker E.A., Welch B. (1990). Role of ferritin as a lipid oxidation catalyst in muscle food. J. Agric. Food Chem..

[90] Ling S.-K., Tanaka T., Kouno I. (2003). Effects of iridoids on lipoxygenase and hyaluronidase activities and their activation by .beta.-glucosidase in the presence of amino acids. Biol. Pharm. Bull..

[91] Shimizu K., Kondo R., Sakai K., Lee S.-H., Sato H. (1998). The inhibitory components from *Artocarpus incisus* on melanin biosynthesis. Planta Med..

[92] Manosroi A., Jantrawut P., Akihisa T., Manosroi W., Manosroi J. (2010). In Vitro anti-aging activities of *Terminalia chebula* gall extract. Pharm. Biol..

[93] Ndlovu G., Fouche G., Tselanyane M., Cordier W., Steenkamp V. (2013). In vitro determination of the anti-aging potential of four southern african medicinal plants. BMC Complement. Med. Ther..

[94] Mandl I., MacLennan J.D., Howes E.L., DeBellis R.H., Sohler A. (1953). Isolation and characterization of proteinase and collagenase from Cl. histolyticum 12. J. Clin. Investig..

[95] R Core Team (2023). R: A Language and Environment for Statistical Computing.

