# Supplementary material for: Antiaging Potential of Lipophilic Extracts of Caulerpa prolifera
Source: Mar Drugs. 2025 Feb 14;23(2):83. doi: 10.3390/md23020083 (PMC11857742; doi:10.3390/md23020083)
Supplement: Supplementary file 1 [file marinedrugs-23-00083-s001.zip › marinedrugs-3422518-supplementary.pdf]

marine drugs

MDPI

Supplementary Material

Antiaging potential of lipophilic extracts of *Caulerpa prolifera*

Gonçalo P. Rosa [1,2], Maria Carmo Barreto [1], Ana M. L. Seca [1,2] and Diana C. G. A. Pinto [2,*]

Table S1. Antioxidant activity by DPPH and ABTS methods of *C. prolifera* extracts.

Sample	DPPH		ABTS	
	% AA*	EC_{50} μg/mL	% AA*	EC_{50} μg/mL
**CP1**	15.3 ± 0.54	>250[a]	31.5 ± 0.11	>250[a]
**CP2**	5.6 ± 0.01	>250[a]	21.9 ± 0.37	>250[a]
**CP3**	37.7 ± 0.37	>250[a]	48.1 ± 1.21	>250[a]
**Trolox**	89.7 ± 0.50	7.3 ± 0.09[b]	87.1 ± 0.95	2.68 ± 0.08[b]

*% Antioxidant activity at 250 μg/mL; In each IC_{50} column, different letters indicate significant differences ($p < 0.05$).

Table S2. ECM degrading enzyme inhibition of *C. prolifera* extracts.

Sample	Elastase		Tyrosinase		Collagenase	
	% Inhibition*	IC_{50} μg/mL	% Inhibition*	IC_{50} μg/mL	% Inhibition*	IC_{50} μg/mL
**CP1**	33.5 ± 0.21	>250[a]	86.4 ± 0.38	31.3 ± 0.37[a]	12.3 ± 0.22	>250[a]
**CP2**	23.1 ± 0.58	>250[a]	36.6 ± 0.19	>250[b]	44.1 ± 0.21	>250[a]
**CP3**	24.8 ± 0.52	>250[a]	37.8 ± 0.23	>250[b]	25.3 ± 0.98	>250[a]
**NMAAK**	95.5 ± 3.96	0.13 ± 0.002[b]	-	-	-	-
**Kojic acid**	-	-	99.2 ± 0.36	1.8 ± 0.13[c]	-	-
**EDTA**	-	-	-	-	96.3 ± 1.2	51.2 ± 0.12[b]

*% Inhibitory activity at 250 μg/mL; In each IC_{50} column, different letters indicate significant differences ($p < 0.05$).

Table S3. Compounds identified by GC/MS on the dichloromethane extract of *Caulerpa prolifera* (CP1).

Rt (min)	Compound	Quantity (mg compound/ 100 g d.w)	Family
18.03	Neophytadiene	52.71 ± 2.55	Diterpene
18.35	Myristic acid	26.15 ± 1.46	SFA
19.24	Phytol acetate	24.87 ± 2.14	Diterpene
22.96	Hexadecadienoic acid	10.37 ± 0.56	UFA
23.11	Hexadecatrienoic acid	14.76 ± 1.41	UFA
23.37	Palmitoleic acid	32.28 2.29	UFA
23.68	Palmitelaidic acid	5.51 0.39	UFA
24.08	Palmitic acid	243.70 6.53	SFA
27.65	Phytol	11.66 0.64	Diterpene
28.64	Linoleic acid	48.22 3.61	UFA
28.80	α-Linolenic acid	30.22 3.11	UFA
29.03	Oleic acid	19.07 0.90	UFA
29.64	Stearic acid	9.74 0.51	SFA
32.67	Oleamide	15.57 1.39	Fatty Amide
32.99	Eicosapentaenoic acid	10.70 0.84	UFA
34.08	11,14-Eicosadienoic acid	8.49 0.34	UFA

34.45	Gondoic acid	7.85 0.37	UFA
36.90	Di-(2-ethylhexyl) phthalate	387.32 28.91	Phtalate
38.41	Erucic acid	13.04 0.91	UFA
41.74	Nervonic acid	21.49 2.01	UFA
42.03	Lignoceric acid	11.07 0.10	SFA
51.29	β-sitosterol	16.96 0.9	Sterol

SFA – saturated fatty acid; UFA – unsaturated fatty acid

Table S4. Mass of fractions obtained from Sephadex column fractionation of CP1.2.

Sample	Mass (mg)	Sample	Mass (mg)
CP1.2.1	0.8	CP1.2.7	11.6
CP1.2.2	97.1	CP1.2.8	19.5
CP1.2.3	54.9	CP1.2.9	13.0
CP1.2.4	53.5	CP1.2.10	4.2
CP1.2.5	58.7	CP1.2.11	14.0
CP1.2.6	25.9	CP1.2.12	4.5

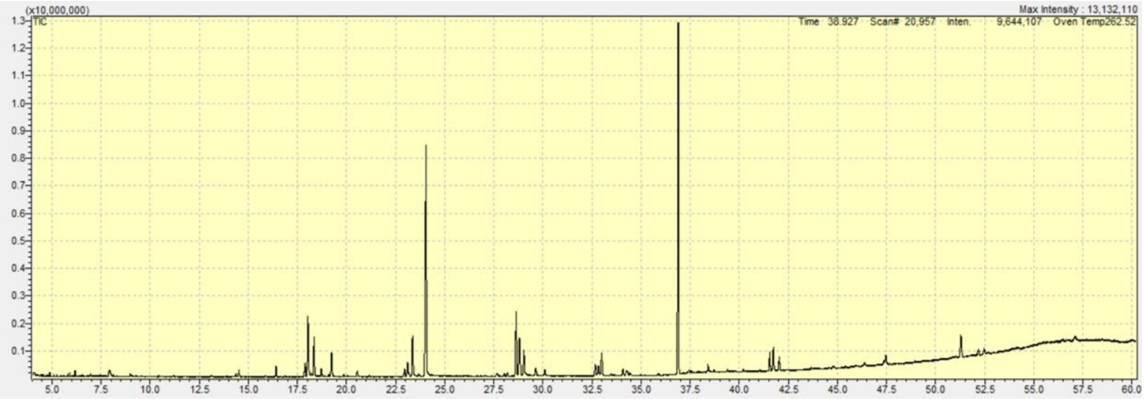

Figure S1. Chromatogram obtained by the GC-MS analysis of CP1.

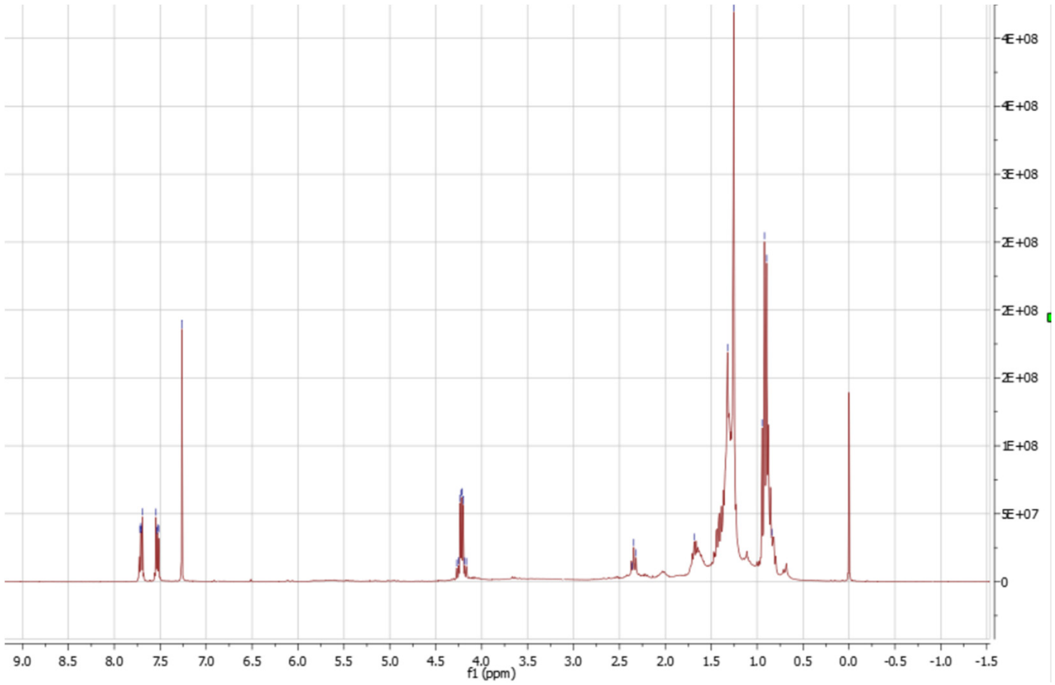

Figure S2. ^{1}H NMR spectrum of fraction CP1.2.8.

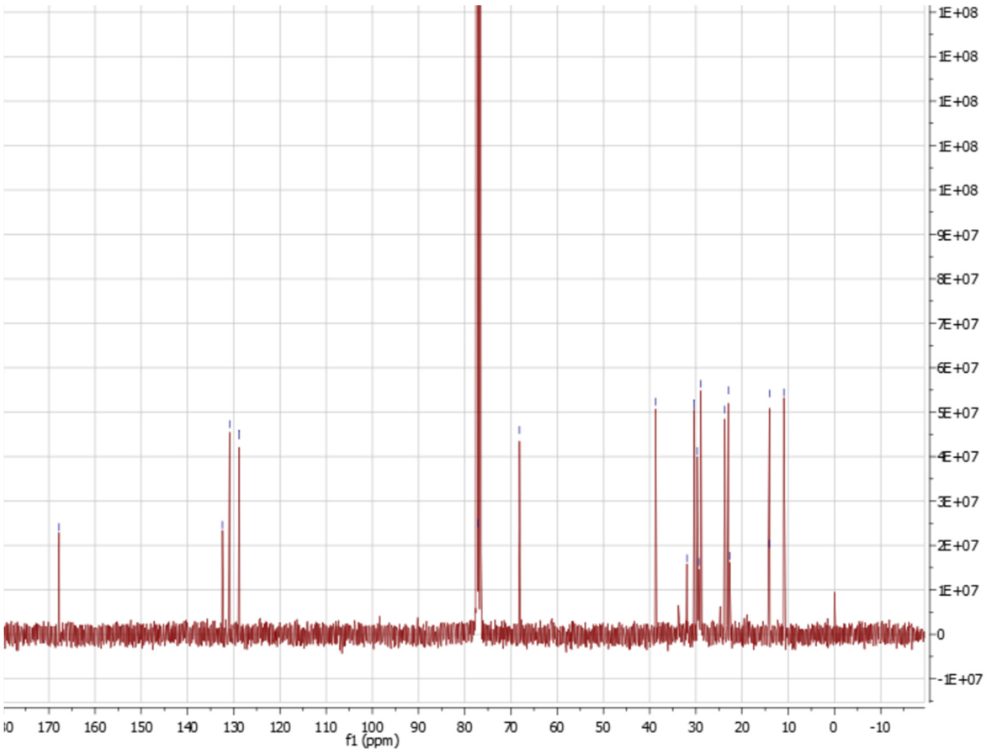

Figure S3. ^{13}C NMR spectrum of fraction CP1.2.8.

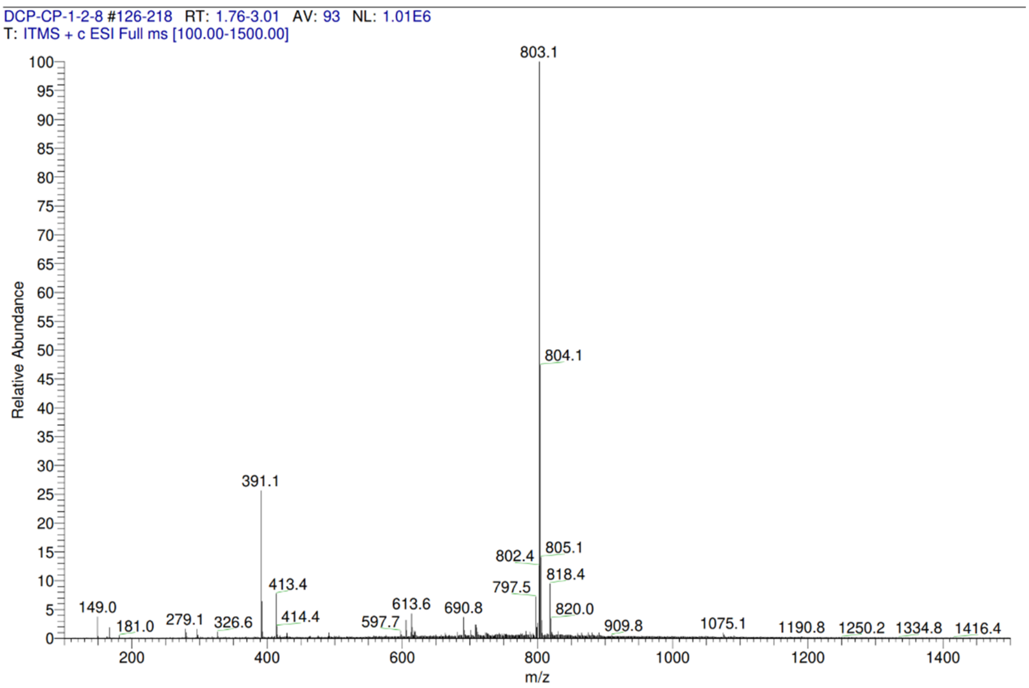

Figure S4. Mass spectra of fraction CP1.2.8.
